# Virtual Simulation in Undergraduate Medical Education: A Scoping Review of Recent Practice

**DOI:** 10.3389/fmed.2022.855403

**Published:** 2022-03-30

**Authors:** Qingming Wu, Yubin Wang, Lili Lu, Yong Chen, Hui Long, Jun Wang

**Affiliations:** ^1^College of Medicine, Wuhan University of Science and Technology, Wuhan, China; ^2^Tianyou Hospital, Wuhan University of Science and Technology, Wuhan, China

**Keywords:** virtual simulation, virtual reality, undergraduate medical education, simulation-based learning, computer simulation

## Abstract

Virtual simulation (VS) as an emerging interactive pedagogical strategy has been paid more and more attentions in the undergraduate medical education. Because of the fast development of modern computer simulation technologies, more and more advanced and emerging VS-based instructional practices are constantly increasing to promote medical education in diverse forms. In order to describe an overview of the current trends in VS-based medical teaching and learning, this scoping review presented a worldwide analysis of 92 recently published articles of VS in the undergraduate medical teaching and learning. The results indicated that 98% of included articles were from Europe, North America, and Asia, suggesting a possible inequity in digital medical education. Half (52%) studies reported the immersive virtual reality (VR) application. Evidence for educational effectiveness of VS in medical students’ knowledge or skills was sufficient as per Kirkpatrick’s model of outcome evaluation. Recently, VS has been widely integrated in surgical procedural training, emergency and pediatric emergency medicine training, teaching of basic medical sciences, medical radiation and imaging, puncture or catheterization training, interprofessional medical education, and other case-based learning experiences. Some challenges, such as accessibility of VS instructional resources, lack of infrastructure, “decoupling” users from reality, as well as how to increase students’ motivation and engagement, should be addressed.

## Introduction

As a positive, safe, and valid reality-based educational approach complementing the traditional teaching methods, simulation is increasingly used in the healthcare areas. Especially in the field of undergraduate education as the cornerstone and starting point of training medical professionals, an extensive body of published studies (1–5) have demonstrated that, when the learners act as they would respond under an environment that they believe to be real, simulation-based learning (SBL) experiences are helpful in integrating theoretical knowledge with practice, and gaining skills necessary for independent practice. As defined as “*a dynamic process involving the creation of a hypothetical opportunity that incorporates an authentic representation of reality, facilitates active student engagement, and integrates the complexities of practical and theoretical learning with opportunity for repetition, feedback, evaluation, and reflection*” (6), simulation used in undergraduate medical education often utilizes goal-based role-plays in the replicated clinical problematic scenarios or case settings in an interactive manner (1–5). Compared with real clinical learning experiences, SBL may be more efficient because the learners intentionally practice skills and higher order thinking. The use of SBL can expose medical students in ethically safe environments without risk of jeopardizing real patients/animals, let them feel safe to make mistakes, and can enhance their confidence while also developing professional knowledge, critical thinking skills, comprehensive decision-making skills, clinical judgment, better clinical preparation, as well as self-efficacy, satisfaction and emotions. Moreover, SBL as a form of education offers repeated practice opportunities especially for less common conditions, and reduces the time consuming to reach professional and clinical competence (1–5). Some previous systematic reviews (4, 7, 8) have shown that medical SBL is effective for the acquisition of clinical skills and contributes to better care of patients. SBL in clinical training such as the use of high-fidelity mannequins, partial task simulators, animal materials or standardized patients, etc. prepares future physicians with communication skills, physical diagnosis, medical interviewing, basic clinical procedures and basic surgical skills in safe and repeated manners, as well as without legal and ethical limits. In pre-clinical undergraduate medical education, the use of SBL serves to reinforce biomedical concepts and other professional knowledge *via* immediate feedback, and introduces low-risk clinical experiential learning amidst a shortage of qualified clinical preceptorships (1). Especially in a resource limited setting, SBL acts as a cost-effective, easily accessible, safe, feasible and promising educational tool that provides more opportunities for medical students to interact with “patients”/“animals” and engage in team work (3, 5). However, multiple factors including the shortages of funding and simulator technologies, the low supply of simulators, the lack of full-time trained staff, the poor motivation and experience limitations of instructors, the time intensive characteristic, etc. have been considered to have negative effects on effective implementation of current simulation-based undergraduate medical education (2, 9, 10).

Driven by the advanced innovations of modern computer and Internet technologies as well as the recent evolution of the medical profession and its teaching dynamics, SBL has conspicuously shifted to virtual platforms, on which simulation-based e-learning is accessible *via* a Web browser, and an upgraded SBL strategy named as virtual simulation (VS) has been produced (11, 12). VS is defined as “*a screen-based simulation where the graphics, sound, and navigation emphasize the three-dimensional (3D) nature of the environment*” (13). The boundaries between the term VS and other technologies such as virtual reality (VR), augmented reality (AR), and virtual standardized patient (VSP), etc. are difficult to define and these terms have been interchangeably used in academic research (13).

During the world wars, VS was initially used in the military area as an aviation training strategy based on a flight simulator. Subsequently, this innovative teaching and learning technological strategy was gainfully applied to more and more technical and workplace training interventions in equipment design, firefighting, law enforcement, lathe operation, vehicle prototyping, crane driving, automotive spray painting, hazard detection, and forestry equipment operation, etc. (9, 12). Sufficient practical learning opportunities are critical for the training of future physicians. However, it is paradoxical that the clinical instructional resources and opportunities for practice are often limited within a university setting due to a large number of undergraduate medical students and finite resources. The positive outcomes of VS in occupational practical training led to its use in undergraduate medical education. Through the re-creation of realistic clinical situations depicted on a computer screen, VS applied in medical teaching based on virtual patients/animals and AR simulations can create an immersive, interactive and risk-free environment for learning practical activities and procedures, thus provide the learners with multiple training possibilities for clinical practices (9, 13). So far, a global interest in VS-based medical teaching programs has been stimulated, and the use of VR, artificial intelligence, machine learning technologies and computer-based serious games is increasingly incorporated into undergraduate medical education practice.

However, because of the fast development of modern computer simulation technologies, more and more advanced and emerging VS teaching instruments, ideas, solutions and practical programs are installed to promote medical education in diverse forms. In order to describe an overview of the current trends in VS-based medical teaching and learning, we here review reports on the practice of using VS tools in medical education at the undergraduate level as documented in recently published literature.

## Methods

In this study, we performed a bibliographic search on the electronic database MEDLINE *via* PubMed using key words “*virtual simulation (VS)* OR *e-simulation* OR *computer simulation* OR *virtual reality (VR)* AND *medical education* OR *medical students*”. Only peer-reviewed articles written in English involving undergraduate medical students and fully published online in recent 2 years (between January 2020 and December 2021) were included. The reviews, technical reports or study protocols without the practical outcomes were excluded. Full-texts of articles were obtained, screened, and underwent quality appraisal independently by two researchers then a consensus reached for included papers. Narrative data were extracted from each included article and downloaded into Excel using the categories listed in Supplementary Tables 1, 2. Data were thematically analyzed. Based the fact that the included studies are pitching at varied levels of outcome measurement, in order to evaluate the outcomes of VS practices, the Kirkpatrick evaluation model (14) was adopted in the present review to aid to segregate, analyze and present the findings of the included articles. Two independent researchers reviewed and grouped data within four levels of the Kirkpatrick model, which are as follow (14): Assessment of learners’ views/satisfaction (Level 1); Change in learners’ views or attitudes (Level 2a); Change in learners’ knowledge or skills (Level 2b); Change in learners’ behavior/practice (Level 3); Change in organizational practice (Level 4a); Change in benefit to patients/health outcome (Level 4b).

## Results

We identified a total of 92 articles reporting the application practice of VS in the undergraduate medical teaching and learning published since 2020 through our search strategy. In Supplementary Tables 1, 2, we summarized the study characteristics and main findings of these previous studies in detail. By and large, the annual numbers of related articles published during 2020 and 2021 were evenly split.

### Distribution of Included Studies

**FIGURE 1:**
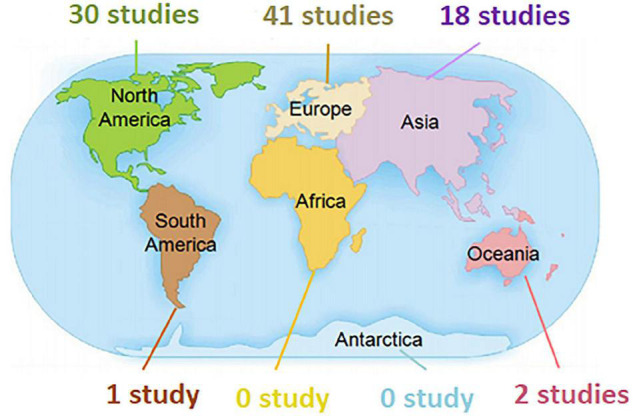
Graph showing the distribution of included studies reporting the use of VS in undergraduate medical education among different continents.

From the 92 published studies, VS was reported to be applied in the educational practice involving undergraduate medical students across 25 countries including the United States [26 studies (15–40)], the United Kingdom [9 studies (41–49)], Germany [7 studies (50–56)], China [6 studies (10, 57–61)], Denmark [6 studies (62–67)], France [4 studies (68–71)], Japan [4 studies (72–75)], Sweden [3 studies (76–78)], Canada [3 studies (79–81)], Netherlands [3 studies (82–84)], Spain [3 studies (85–87)], Australia [2 studies (88, 89)], Singapore [2 studies (90, 91)], Korea [2 studies (92, 93)], Finland [1 study (94)], Italy [1 study (95)], Ireland [1 study (96)], Colombia [1 study (97)], Pakistan [1 study (98)], Thailand [1 study (99)], Iran [1 study (100)], Poland [1 study (101)], Mexico [1 study (102)], Norway [1 study (103)], Saudi Arabia [1 study (104)], and Switzerland [1 study (105)]. The distribution of included studies among different continents was shown in Figure 1. Results showed that nearly half of studies (45%) were from Europe; one third (33%) from North America; 20% from Asia, while none was from Africa.

### Virtual Simulation Tools Used in Undergraduate Medical Education

Despite the diversification of virtual simulators/platforms/systems used in undergraduate medical education, we found that 48 (52%) studies (18, 22, 23, 27–31, 33–35, 37–39, 42, 44–48, 50, 52, 54, 57–67, 71, 72, 75, 78, 81–84, 93, 96, 99, 101, 103, 105) reported the immersive VR application, which is characterized by the use of VR equipment consisting of head-mounted displays (headsets or goggles) and/or hand controllers. This finding suggested that VR might be a typical and popular representative of modern VS technology used in medical education. Moreover, only one third of (31) articles (15, 16, 22, 26, 31–33, 38, 47, 48, 52, 53, 56, 58, 60–62, 64, 65, 67, 71, 73, 75, 76, 78–80, 95, 96, 101, 105) included in this review were based on the commercially available or free VS softwares/platforms, the rest used the self-developed ones.

### Kirkpatrick’s Four-Level Evaluation of Included Studies

All the included studies involved the outcome evaluation that can be mapped to Kirkpatrick’s four-level model. Using the Kirkpatrick’s evaluation model to structure the analysis of evidence from these studies, a lens was afforded for integrating the findings to identify that a vast majority of included studies (67 studies; 73%) evaluated at Level 2b of the Kirkpatrick’s model, included the changes in learners’ knowledge or skills. In addition, 23 studies included the Kirkpatrick Level 1 evaluation of learner satisfaction, and two studies reported the changes in learners’ views or attitudes (Level 2a). No study met the Level 3 (practice change) and Level 4 (health outcome) of Kirkpatrick’s model.

These findings suggested that evidence for educational effectiveness of VS in medical students’ knowledge or skills was sufficient. There was no study particularly presenting the students’ performance change in clinical practice or the possible benefit to patient/health outcome. More pedagogical research might be merited to inform effective evaluation of the effect of VS used in undergraduate medical education on learners’ behavior/practice as well as its clinical effectiveness.

### Virtual Simulation-Based Learning Contexts and Practical Aspects

**FIGURE 2:**
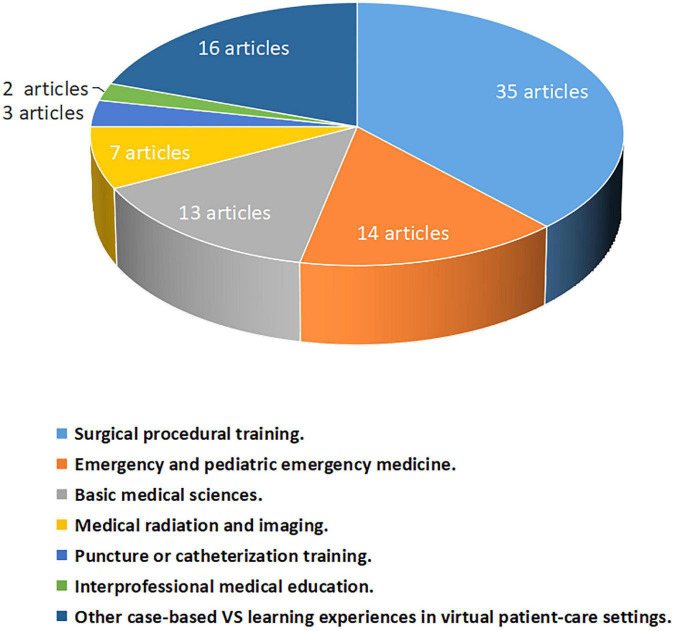
Graph showing the distribution of 92 articles included in this review among learning contexts and practical aspects of undergraduate medical education.

In spite of the varied study purposes of included articles, these previous attempts at least open up possibility and suggest potential for VS application in undergraduate medical education. Based on the studies included for review, we summarized that, in the recent 2 years, VS has been integrated in the following learning contexts and practical aspects of undergraduate medical education (Figure 2).

#### Simulation for Surgical Procedural Training

Of the included 92 papers, 38% (35 paper) reported the integration of VS in surgical training for medical undergraduates, among which 12 studies focused on the instructional application of virtual endoscopic [including laparoscopic (31, 36, 37, 56, 73, 76, 78, 98), arthroscopic (26, 47, 48), and otoscopic (53)] simulators; 7 studies were for learning procedures or concepts of orthopedic and bone surgery (32, 33, 35, 45, 57, 66, 70); 5 studies were based on VS system or platform as a primary mode of teaching neurosurgical procedures, neuroanatomy and pathologies (22, 28, 71, 80, 81); 4 papers (38, 61, 75, 84) reported the exposure of medical undergraduates as novice surgeons to the robotic surgery simulators; 2 studies conducted by the same team (64, 65) explored the VR simulation-based training in Cochlear Implant surgery; the other 2 were for learning basic motor skills in liver surgery (50, 52); 1 in minimally invasive surgery (97), and 1 in vitreoretinal surgery (67). In addition, Fukuta et al. (46) generated a virtual operating theater orientation to improve knowledge and confidence of medical undergraduates. Except five validation studies of virtual simulators (26, 47, 53, 65, 81) in which the undergraduates acted as the novice group for comparison with the skilled group, the findings of all the other included studies positively supported the usability and its feasibility of integrating VS in surgical training.

As one of crucial links in medical education, competence-based training of surgical skills is important from the undergraduate phase. Sufficient and high-quality training, deliberate practice, as well as mastery of surgical techniques and instruments are imperative for future surgeons. However, the high risks of injury, the slow learning curves, as well as the limited opportunities to practice, etc. are challenging the modern surgical training. Compared with the traditional master-apprentice surgical education, VS-integrated surgical training can provide desirable alternative allowing an active, independent, repeated and safe learning for students to become familiar with procedures, instruments, and equipment before performing surgeries on patients. In particular, VS-based learning has been found to be conducive to the development of complex psychomotor skills, such as hand-eye coordination that endoscopic and robotic surgery sets particular demands on (64, 65, 97). A recently published article conducted by Petersen et al. (67) found no positive skill transfer from basic skills pre-training in a VR vitreoretinal simulator to the procedure-specific modules, suggesting that, compared than spending valuable training time on basic skills VS pre-training, proceeding directly to VS-based training of procedures was more meaningful for learners.

#### Simulation for Emergency and Pediatric Emergency Medicine Training

The second focus of VS-integrated learning contexts in undergraduate medical education was emergency and pediatric emergency medicine training, with 14 articles (10, 16–18, 29, 30, 34, 39, 51, 55, 68, 79, 93, 101) published recently. Among them, eight studies (10, 16–18, 30, 34, 39, 68) used VS-based teaching in pediatric emergencies. The VS simulators used in these studies were mainly based on virtual patient cases, and simulated the clinical critical events.

Emergencies especially pediatric and neonatal emergencies are relatively rare, but potentially catastrophic. However, the life-saving emergency management skills are difficult to master, and accordingly used uncommonly enough to make skill acquisition a challenge (18). In order to provide a safe environment for unlimited exposure to rare clinical events and training in high-risk procedures, SBL has long been considered as a cornerstone of emergency medicine training (16).Unfortunately, as a frequent approach to traditional SBL, standardized patients for emergencies especially for pediatric emergencies are not on option. Many available mannequin-type patient simulators cannot fully display the realistic conditions for training and assessment of competency, as well as critical physical examination findings, such as work of breathing and mental status, in clinical emergency events (30). The above studies showed the integration of VS in emergency medicine training provided promising zero-risk training for undergraduates. In particular, seven studies (18, 29, 30, 34, 39, 93, 101) used the VR simulation systems consisting of VR headsets or goggles to realistically and immersively replicate clinical settings and findings, allowing students to deliberately practice and receive vivid feedback on their assessment.

#### Integration of Virtual Simulation in Teaching of Basic Medical Sciences

As the foundation of medical practice, basic or pre-clinical sciences are considered as the “*core component*” for clinical education. The active and efficient learning experiences in pre-clinical years for in-depth mastery of the basic medical knowledge shape up clinically competent and scientifically grounded physicians (106). Modern pre-clinical curricula lay emphasis on practical-oriented, laboratory-based hands-on training. However, most basic medical curricula are highly information-intensive. Especially facing the reduction of contact hours and limited resources, the use of VS in teaching of basic medical sciences has been paid more and more attentions (106).

We found that, since 2020, there were 13 articles reporting the application of VS for teaching basic medical sciences, including anatomy (23, 42, 44, 49, 59, 60, 69, 72, 82, 104), physiology (92, 94) and pharmacology (19). Obviously, VS-enhanced anatomy training is a focused program as a powerful supplement in conventional anatomy teaching settings. Traditional methods for understanding anatomy include lectures, textbooks, cadaveric dissection, the viewing of prosections, illustrations, photographs, physical models, etc. (42). However, the teaching efficiency may be lacking because of traditional 2D images, as well as limited and expensive cadaver or mannequin resources. As the revolution of anatomy education through digital media, VS has been demonstrated to provide vivid and dynamic imagery that the students can interact with an active learning experience without having to study in an anatomy laboratory (23, 42, 44, 49, 59, 60, 69, 72, 82, 104). Especially for some topics that are challenging to teach because of complex 3D nature, VS-integrated teaching facilitated the 3D spatial perception of anatomy, and helped the students learn more efficiently. Among the included 10 articles reporting VS application in anatomy education, 6 (42, 49, 59, 60, 72, 104) used virtual simulators to help students see the details of muscle and bones; 1 (44) for neuro-anatomy; 1 (23) for cerebrovascular anatomy; and 1 (69) for prostate. The other study conducted by Bogomolova et al. (82) developed a virtual 3D assessment scenario for undergraduate anatomical education. Similarly, the other reports about teaching practices on the use of VS in the pre-clinical phase were in the fields of neurophysiology (94), cardiac physiology (92) and psychopharmacology (19), respectively.

#### Virtual Simulation-Integrated Learning in Medical Radiation and Imaging

Medical radiation and imaging data such as CT, MRI, and ultrasound are indispensable for the clinical diagnosis. Undergraduate medical education is responsible for pedagogical preparation of medical radiation practitioners. However, being limited by radiation safety reasons, the exposure of medical undergraduates to clinical imaging teaching materials is generally insufficient (88, 96). With the technological development of digital radiographic reconstruction with geometric as well as density characteristic accuracy (107), more and more VS computer software programs have been designed and used in undergraduate medical education, allowing students gain more hands-on experience and develop their clinical skills without worrying about exposing to any unnecessary radiation. In the recent 2 years, we found five studies (20, 85, 86, 88, 96) used VS serious games or systems simulating a radiologist’s practice in the real world among medical undergraduates for radiology learning. The results collectively showed that, for medical undergraduates, the integration of VS as a valuable learning resource had the potential to improve preparation for the clinical environment and increase student confidence (20, 85, 86, 88, 96).

The other two studies (58, 62) demonstrated the effectiveness of VR learning integrated in ultrasonography training for improving students’ ultrasound skills, and students reported they wanted more VS learning. As the VR educational tool is not as space demanding or as expensive as ultrasound simulators, it could be appealing for medical schools with limited resources for basic ultrasound training (62).

#### Simulation for Puncture or Catheterization Training

Mastering the skill and procedures of puncture or catheterization is essential across many medical specialties. However, as an invasive operation that may cause patients discomfort and have the risk of complications, puncture or catheterization has been considered as a challenge for medical training (63, 99). In the recent 2 years, three published articles reported the application of VR in VS training among medical undergraduates for lumbar puncture (25), ultrasound-guided peripheral venous catheter placement (63), and endotracheal intubation (99), respectively. The results collectively suggested that, as a teaching method well-received by students, VS training can engage learners, develop their practical competencies and proficiency in performing procedures under safe and controlled environments, facilitate spatial recognition and anatomic visualization, thus enhance medical education and skills training (25, 63, 99).

#### Offering Opportunity for Quality Interprofessional Medical Education Delivery

As a critical component of modern patient-centered healthcare practices, the interprofessional team–based model of care in which multiple healthcare professionals including physicians, nurses and pharmacists, etc. work together has been associated with enhanced patient satisfaction and better quality in patient care (108, 109). It has been well-accepted that interprofessional team training should commence at the undergraduate level and continue into clinical practice (91). Simulation-based experiential learning methods represented by role-play have been widely used in undergraduate medical education, and proven to be effective for interprofessional team training (91, 108, 109). Nevertheless, traditional simulation-based interprofessional education in undergraduate stage is confronted with challenges, such as difficulties in getting together different professions of healthcare students as well as the lack of simulation facilities and interprofessional facilitators (90, 91). Importantly, based on its multi-user feature, VS offers an opportunity for healthcare undergraduates from different professions and different institutions to efficiently participate in interprofessional education. In 2020, Liaw et al. (91) reported an integration of computer-based VR into interprofessional team training curriculum among undergraduate medical and nursing students. No difference between virtual and live simulations was found in terms of students’ attitudes toward teamwork and communication skill performances, suggesting the potential use of VR to substitute conventional simulation training in interprofessional education. Subsequently, under the background of COVID-19 pandemic, the same study team applied the Internet-based 3D virtual world mimicking the real hospital environment for VS-integrated interprofessional training, and geographically dispersed undergraduate students from six different healthcare professions (medicine, nursing, pharmacy, occupational therapy, physiotherapy, and medical social work) experienced this VS-based learning using their own avatar roles (90). Results also showed that this immersive and realistic VS tool offered opportunity for high-quality interprofessional medical education delivery.

#### Other Case-Based Virtual Simulation Learning Experiences in Virtual Patient-Care Settings

In addition to the above learning contexts and practical aspects, the remaining 18 articles (15, 21, 24, 27, 40, 41, 43, 54, 73, 77, 83, 87, 89, 95, 100, 102, 103, 105) reported the integration of VS into other case-based learning experiences in virtual patient-care settings. Despite the diversity of virtual patient systems and clinical scenarios, these studies generally showed that VS-integrated case-based learning as a feasible teaching approach (54) could result in students’ learning gains, retention of information, and transfer of knowledge to clinical application (89, 95, 100, 102), help future physicians improve diagnostic accuracy thus enhance the clinical reasoning teaching (15, 27, 43), extend students’ preparedness level for their future clinical experiences (40, 83), facilitate empathy (24), cultural competence (77) and comprehensive clinical skills such as communication-based skills (21), clinical decision-making skills (78) within undergraduate medical education, and improve students’ confidence in managing clinical scenarios (41), thus was highly received by students (89, 95, 105).

Especially during the COVID-19 pandemic, this pedagogical modality avoided training interruption and was highly valued (41, 87, 95, 105). Due to risk of COVID-19 exposure and required social distancing, the students’ clinical placements, face-to-face teaching and practical/lab sessions have all been limited even canceled in the pandemic situations, and a sudden and complete disruption in medical education has occurred (15, 16, 106, 110). The restrictions due to COVID-19 raise the need for innovative medical VS teaching methods, which provide educational contents in a learning environment where lecturers and students separated by space or time or both (41). However, the sudden outbreak of COVID-19 poses the difficulties in altering medical training modality during an extremely short period of time. In this situation, VS-based learning that has been widely adopted in medical schools is considered as a prompt turning point in medical education to overcome the educational gap due to COVID-19 (16, 17, 41, 79, 111). Through the application of VS, it is potential to digitally reconstruct the clinical environment, simulate the clinical learning and ensure the continuation of practical examinations, in spite of widely dispersed student or faculty placements (112). After outbreak of COVID-19, De Ponti et al. (95) conducted a questionnaire-based survey among 115 pre-graduated medical students, and showed that 97 students (84%) considered the future use of VS training useful in addition to the traditional apprenticeship at patient’s bedside, suggesting medical students’ appreciation for the application of VS in post-pandemic medical education. The integration of emergent technology represented by VS into medical curriculum has been considered as an indispensable component of the transformative change and post-COVID undergraduate medical education to keep the medical education on stream (106). Especially in the face of the current ongoing COVID-19 crisis, VS could act as a flexible teaching and learning modality in response to further pandemic waves.

In addition, the students’ performance on learning tasks can be well-assessed using VSPs (21), or a computer-based case simulation objective structured clinical examination (OSCE) (87). However, a study (103) compared a fully immersive, interactive, multiplayer VR application in the group self-practice of systematic clinical observation using the airway, breathing, circulation, disability and exposure (ABCDE) approach to the physical equipment, and the results showed that group self-practice of the ABCDE approach in VR application was non-inferior to practice with physical equipment. Therefore, further practice and research on the integration of different virtual patient VS systems in case-based learning experiences under various clinical scenarios might be required to identify the role of VS in undergraduate medical education.

## Discussion

This study reviewed the recent practice of VS in the undergraduate medical teaching and learning reporting in 92 articles since 2020. Evidence for educational effectiveness of VS in medical students’ knowledge or skills was sufficient as per Kirkpatrick’s model of outcome evaluation. We found that VS was applied in the educational practice involving undergraduate medical students across 25 countries. However, an overwhelming majority of (97%) involved studies were form Europe, North America, and Asia. This regional bias might be due to the uneven distribution of digital medical education resources across the world, which would influence the local medical students’ access to education in underdeveloped areas. However, the highly shareable feature of digital resources has been considered to provide an opportunity to address the need for a fair learning system for medical students and promote equity in medical education globally (113, 114). Even in resource limited settings, the application of VS educational systems/platforms could help to promote medical learning by reducing instructor costs and laboratory materials. Along with the advancement and expansion of computer technology, VS has been believed as a less expensive and more accessible alternative for undergraduate medical education, allowing for its wide application in low-and-middle-income countries (85, 115). So far, increased availability and affordability of technology-based commercial platforms, such as Google, Apple, and Microsoft, allow any medical educational institution to share VS resources, or engage in research and development of VS projects to improve their efficiencies within curricula. For example, as an international virtual community with more than 1,500 million square meters allowing tens of thousands of users connected at the same time around the world, *Second Life*^1^ created by Linden Laboratories in 2003 has become the most active virtual world in higher education. Currently, hundreds of universities around the world have used it to support teaching and learning activities. As an educational tool, *Second Life* has been dedicated to the training of medical undergraduate students in areas such as radiology (85, 116) and anatomy (86, 115, 117). Therefore, once being promoted to more medical schools around the world, VS learning produces based on platforms such as *Second Life* will help promote greater equity in global medical education. Similarly, the University of Southern California, United States, developed a freeware virtual patient community, the *University of Southern California Standard Patient Studio* platform, with funding from the Department of Defense. This platform allows for the creation of personalized VSP software for different teaching and learning purposes, and has been shared by other US medical schools (19). In addition, a company (Oxford Medical Simulation) is offering a VR medical education platform where undergraduate students can take medical histories, examine, diagnose and treat digitally simulated patients within a virtual clinical environment (118). Nowadays, *Human Patient Simulators and Virtual Reality Laparoscopic Trainers* have been well-developed by manufacturers and are available on the market (120, 121). Therefore, professional teaching materials that were previously limited to certain settings or world-renowned medical schools are now being released on VS-based platforms that can be employed by any user across institutions, areas and countries (122). In China, a profile file-sharing website named the *National Virtual Simulation Experiment Teaching Project Sharing Platform*^2^ is readily available with minimal setup and free access, in which the abundant medical VS teaching resources contribute greatly to the nationwide equity in undergraduate medical education. To date (1 October 2021), the VS teaching resources in the areas of pre-clinical and clinical medicine have been visited near 350,000 times. If the language barrier can be overcome, these VS medical teaching materials may be shared by more medical schools around the world.

Lack of infrastructure such as computer hardware and network has been considered as one of major challenges to establishing VS-integrated curricula (90). It has been found that computer self-efficacy might affect the learners’ willingness to adopt the VS as part of learning (90). For remote VS experiences, the Internet connection bandwidth could impact the learning experience, and contribute to the technical issues (90). Here, we found the immersive VR approach is the currently popular VS tool used in undergraduate medical education, the application of which was reported in half (52%) involved studies. However, the cost and the provision of satisfactory VR equipment such as head-mounted displays and hand controllers might limit final implementation of VR-integrated educational practice in low- and middle income countries. Some studies (75, 105) showed that, during the VR-simulation, “*visually-induced motion sickness*” shown as nausea, headache, blurred vision, and dizziness might cause a disturbing impact on some learners at the physical level. Moreover, because the headsets and other VR equipment are used communally among undergraduate medical students, it should be necessary to disinfect the VR simulation tools for public use between uses, especially during the COVID-19 pandemic (105). In addition, we found only one third of involved studies used the commercially available or free-accessible VS softwares/platforms for the undergraduate medical teaching and learning. Actually, designing and developing new VS instructional simulators or creating VS educational scenarios require significant inputs of time, funds and effort for educators. Currently, due to the excessive rapid change of VS technology, there are no standardized or well described VS design approaches (13). Continued back-and-forth collaboration among educators, clinicians and engineers in design and development teams is critical to advancing the establishment and implementation of VS-integrated undergraduate medical training (57). It had been estimated that at least 1 year need to be spent to achieve an acceptable VR simulator for medical undergraduates (57). These barriers might provide incentive for educators to centralize VS medical educational resources. However, only through increasing availability and awareness of developed VS instructional tools among larger audiences, individual costs can be shared and the above barriers will be minimized. If possible, freely sharing online VS educational resources may help equalize global medical education.

Recently, VS has been widely integrated in various learning contexts and practical aspects of undergraduate medical education, including surgical procedural training, emergency and pediatric emergency medicine training, teaching of basic medical sciences, medical radiation and imaging, puncture or catheterization training, interprofessional medical education, and other case-based learning experiences. Among them, the most focused field of study is the application of VS tools in training of surgical skills; however, more attempts are needed to apply VS in interprofessional medical education and training of puncture/catheter skills. Generally, VS has been well-accepted as a valuable pedagogical approach for undergraduate medical education. Through providing computer-generated immersive learning scenes being highly realistic, diversified, dynamic and customized, VS used in undergraduate medical education offers an opportunity for students to achieve first-person experiences in life-like and complex clinical scenarios that they may not normally be exposed to, or when it is hard to access patients, and makes learning effective and appealing to students. However, content provided on a screen using a digital device might “decouple” users from reality. Hands-on experience is essential for medical students to master clinical skills, for example, surgical techniques. Several previous studies (18, 123) have suggested that improved performance in the VS environment might not always transfer to the clinical setting. Therefore, VS is insufficient to replace hands-on experiential practice for medical students to master clinical skills, which might be another important challenge. The current VS simulators act as only part of the medical comprehensive training to supplement the hands-on experience but not the only training technique. In addition, an interesting study (86) explored the impact of compulsory participation on the VS learning experiences of medical undergraduates. The results showed that the learning performance and acceptance of VS technology were lower with a compulsory participation, and the opinion toward VS-based study was even worse if dropouts were not allowed. Therefore, learning in VS environments should be voluntary (86). And how to increase students’ motivation and engagement is an important issue for medical educators to achieve the effective integration of VS into undergraduate education.

## Author Contributions

QW and JW substantially contributed to the conception and the design of the work. All authors have contributed to the interpretation of the data and the drafting of the work, they revised several versions of it and have approved the submitted version.

## Conflict of Interest

The authors declare that the research was conducted in the absence of any commercial or financial relationships that could be construed as a potential conflict of interest.

## Publisher’s Note

All claims expressed in this article are solely those of the authors and do not necessarily represent those of their affiliated organizations, or those of the publisher, the editors and the reviewers. Any product that may be evaluated in this article, or claim that may be made by its manufacturer, is not guaranteed or endorsed by the publisher.

## Footnotes

1
http://secondlife.com/

2 http://www.ilab-x.com/ (in Chinese).
