# Supplementary material for: Virtual Simulation in Undergraduate Medical Education: A Scoping Review of Recent Practice
Source: Front Med (Lausanne). 2022 Mar 30;9:855403. doi: 10.3389/fmed.2022.855403 (PMC9006810; doi:10.3389/fmed.2022.855403)
Supplement: Supplementary file 1 [file Data_Sheet_1.pdf]

Table S1. Summary of study characteristics and main findings of included studies published in 2020 (n=42).

Author (year)	Country	Purpose	Virtual simulator/ platform /system	Study design	Included population	Outcomes measurements	Main findings	Kirkpatrick level
Björn *et al.*, 2020 [94]	Finland	To explore how the introduction of a personal computer (PC)-based electroencephalography (EEG) simulator in a higher education neurophysiology course would enhance students' acquisition of practical skills and theoretical knowledge.	PC-based simulator for EEG electrode placement with a logical (human-friendly fuzzy feedback) system.	Mixed-methods intervention Study	A total of 35 students in the clinical neurophysiology course at two universities	● A pretest– posttest design to evaluate theoretical knowledge about EEG methods and neurophysiology.<br>● The practical skill of EEG electrode placement.<br>● Self-reported simulation experience and students' diary entries.	● Students who studied using the EEG simulator exhibited greater knowledge of the EEG method than students who did not use simulation.<br>● Based on the learning diaries, 45.7% (16/35) of the students indicated that PC-based simulations are generally useful in education.<br>● In addition, one-half (18/35, 51.4%) of the students indicated that they would be ready to use their own time to practice the EEG method and electrode placements using a PC-based simulator.	2b
De Ponti *et al.*, 2020 [95]	Italy	To assess medical students' perception on fully online training including simulated clinical scenarios during COVID-19 pandemic.	Simulated clinical scenarios (Body Interact™) of 21 patient- based cases	A intervention Study	Sixth-year medical students (n=115)	A 12-item anonymous questionnaire on a voluntary basis to rate the training quality.	● 104 (90%) gave positive evaluation to VR training and 107 (93%) appreciated the format in which online training was structured.<br>● The majority of participants considered the platform of VR training realistic for the initial clinical assessment (77%), diagnostic activity (94%), and treatment options (81%).<br>● 97 (84%) considered the future use of this VR training useful in addition to the apprenticeship at patient's bedside.<br>● 32 (28%) participants found the online access difficult due to technical issues.	1

Author (year)	Country	Purpose	Virtual simulator/ platform /system	Study design	Included population	Outcomes measurements	Main findings	Kirkpatrick level
Kolla *et al.*, 2020 [42]	United States	To assess and compare the utility of VR in anatomy education with traditional teaching methods including lecture and cadaveric dissection.	A self- developed VR anatomy-training module including the VR headset and 2 controllers, one for each hand.	A intervention study	Twenty-eight first-year medical students	Survey questions	● Approximately 80% of our subjects reported VR technology was helpful for learning anatomy and 90% reported it was helpful for learning the names and locations of 3D anatomical structures.<br>● The large percentage (97%) of subjects reporting VR more helpful than lecture.<br>● 5/28 (17.9%) of the subjects reported VR to be "much better" and 15/28 (53.6%) of the subjects reported VR to be "somewhat better" than dissection for learning 3D anatomical relationships, suggesting that they held value to cadaveric dissection for learning 3D anatomical relationships.	1
Oussi *et al.*, 2020 [76]	Sweden	To investigate the impact of personal computer–gaming experience, visuospatial skills, and repetitive training on laparoscopic simulator performance and specifically on the constituent parameters of the simulator score.	Minimally Invasive Surgical Trainer– VR simulator (MIST-VR, Mentice, Gothenburg, Sweden)	A intervention study	Fifty-seven medical students during their surgical semester	● A visuospatial test.<br>● Performance scores measured by the built-in software of the MIST-VR simulator.<br>● A questionnaire regarding personal computer–gaming experience.	● There was an association between personal computer–gaming experience and the coordination parameters of the score (p = .0047-0836).<br>● Visuospatial ability correlated with Minimally Invasive Surgical Trainer simulator performance but was abolished with repetitive training.	2b
Alvarez-Lopez *et al.*, 2020	Colombia	To evaluate a simulation instrument —SIMISGEST-VR—	SIMISGEST-VR (a self-developed portable Simulator	A prospective observation	Medical undergraduate students (n=100,	Task metrics including time (velocity), efficiency of movement	The tasks for learning basic motor skills in minimally invasive surgery demonstrated high internal consistency and high test-retest	2b

Author (year)	Country	Purpose	Virtual simulator/ platform /system	Study design	Included population	Outcomes measurements	Main findings	Kirkpatrick level
[97]		and to document the sources of validity evidence for task scores, relations to other variables, internal structure, consequences of testing, and response process.	of Minimally Invasive Surgery mediated by Gestures - Virtual Reality)	al validity study	reference group), surgical residents (n=20), and experts in minimally invasive surgery (n=28) took part in the study.	for the right and left hands, economy of diathermy, error and accuracy (penalty), and final score provided by the simulator	reliability among undergraduate students when assessing the task scores.	
Mirchi *et al*., 2020 [80]	Canada	To introduce a new framework using explainable artificial intelligence for simulation-based training in surgery, and validate the framework by creating the Virtual Operative Assistant, an automated educational feedback platform	The Virtual Operative Assistant, a linear Support Vector Machine (SVM) developed in a previous study to evaluate a VR brain tumor resection task on the NeuroVR simulation platform (CAE Healthcare, Montreal, Quebec, Canada)	A framework validation study	Twenty-eight skilled participants (14 staff neurosurgeons, 4 fellows, 10 PGY 4-6 residents) and 22 novice participants (10 PGY 1-3 residents, 12 medical students)	Two-hundred and seventy metrics of performance created using the data recorded by the simulator.	● The Virtual Operative Assistant successfully classified skilled and novice participants using 4 metrics with an accuracy, specificity and sensitivity of 92, 82 and 100%, respectively.<br>● Initial validation with a linear SVM revealed the feasibility of using the framework to build an automated and objective feedback platform for a complex neurosurgical task.	1
Rakofsky *et al*., 2020	United States	To develop a virtual standardized patient-based	A virtual standardized patient	A comparative study	The analyzed sample included 6 fourth-year	● A novice-expert performance comparison.	● Total mean scores on the exam differed significantly across all the learner groups in a step-wise manner from students to faculty (F	2a

Author (year)	Country	Purpose	Virtual simulator/ platform /system	Study design	Included population	Outcomes measurements	Main findings	Kirkpatrick level
[19]		assessment simulator to address biases and practical limitations in existing methods for evaluating residents' proficiency in psychopharmacological knowledge and practice.	(VSP)–based psychopharmacology simulator created through the University of Southern California Standard Patient Studio platform, a freeware virtual patient community.		medical students (15% of the total sample), 5 PGY-1 residents (12.5%), 10 PGY-2 residents (25%), 6 PGY-3 residents (15%), 8 PGY-4 residents (20%), and 5 faculty members (12.5%).	● A correlational analysis of simulator performance with the PRITE Somatic Treatments subscale score. ● Post-test surveys.	= 6.10, p = 0.0001). ● Total mean scores by residency class correlated with PRITE Somatic Therapies subscale scores ($p < 0.01$). ● The post-test survey mean Likert results ranged from 3.33 ± 1.20 to 4.4 ± 0.79, indicating neutral to favorable responses for use of the simulator.	
O'Rourke *et al.*, 2020 [21]	United States	To compare the emotional experiences of individuals who interacted with real SPs (rSPs) and computer-based virtual standardized patients (vSPs) .	A single SP was used for this study. The AI module had been preprogramed to mimic the unique body language and facial expressions of the SP to make the mannerisms of the SP maximally similar across the rSP and vSP conditions.	A randomized parallel-group study	Sixty medical student participants were randomly assigned to deliver bad news to an rSP (n=28) or vSP(n=32).	Salivary cortisol and a self-report measure of mood-the Profile of Mood States, Second Edition (POMS 2)-were gathered before and after delivering the bad news.	● Participants in both conditions reported increased negative emotionality on the POMS 2 following the SP interaction. There were no significant between-group differences on the POMS 2 or salivary cortisol concentration following the SP interaction. ● Ratings by the SP and independent evaluators indicated that participants performed similarly on most interpersonal dimensions, except tone of voice. ● Participants perceived the vSP as less realistic than the rSP.	1

Author (year)	Country	Purpose	Virtual simulator/ platform /system	Study design	Included population	Outcomes measurements	Main findings	Kirkpatrick level
Galvez *et al.*, 2020 [23]	United States	To evaluate a case-centered, immersive, easily customizable VR task that supported students with learning peripheral and collateral circulation, anatomical features that are not easily observed in cadavers.	The VR version of Organon (commercially available anatomy program; https://www.3dorganon.com/) and Vive headsets (commercially available VR hardware; https://www.vive.com/us/).	A intervention Study	32 students	● Weekly NBME-style quizzes (two questions the week of the activity and one the following week).<br>● Survey<br>● Focus group	● Overall student performance on relevant items on weekly NBME-style quizzes was 96% correct, compared with 75% nationally.<br>● A cross-correlational analysis of Likert-type responses demonstrated a high correlation between the activity as an effective approach to learning and intent in using VR for independent study (R = 0.7).<br>● There was a strong correlation between students that indicated they would use VR for independent study and a desire for this activity to be offered again (R = 0.7).<br>● This proved to be both enjoyable for the students and successful in delivering the desired educational content.	2b
Roehr *et al.*, 2020 [25]	United States	To evaluate the effectiveness of VR training and a student-led simulation module in preparing medical students to perform a lumbar puncture.	The Simulation Curriculum Director of the College of Medicine designed a VR lumbar puncture training module from a lumbosacral MRI using Arivis InView^{R} patent	A comparative Study	Twenty-five medical students were randomly distributed into the VR group (n = 13) , or the curriculum's standard student-led procedural instruction group (n = 12).	Pre- and post-intervention videos recorded as they performed the lumbar puncture procedure were scored using a critical action checklist in conjunction with time needed to complete the procedure to evaluate proficiency.	● Assessing overall post-intervention performance, both groups showed improvement in aggregate score ($p < 0.001$) and time required to complete ($p = 0.002$) the lumbar puncture.<br>● Following interventions, the student-led group improved over the VR group in a variety of metrics. The student-led group increased their aggregate score by 3.49 and decreased their time to completion by 34 s over the VR group when controlling for baseline measures.	2b

Author (year)	Country	Purpose	Virtual simulator/ platform /system	Study design	Included population	Outcomes measurements	Main findings	Kirkpatrick level
			pending technology (AG Imaging Science Unit, Phoenix, AZ).					
Kim *et al*., 2020 [27]	United States	To evaluate and compare the diagnostic accuracies and preferences of various display systems, including the conventional 2D display and a novel group VR software, in group discussions of congenital heart disease (CHD).	A self-developed group VR software, Cardiac Review 3D using the Unity engine.A mobile head-mounted display, Gear VR (Samsung Electronics Co Ltd).	A intervention Study	A total of 22 trainees consisting of 1 1st -year, 10 2nd-year, 4 3rd-year, and 1 4th-year residents and 6 medical students were formed into groups of 4-5 participants. Each group discussed 3 diagnostic cases of CHD with varying structural complexity using conventional 2D display and VR software.	• The discussion time, diagnostic accuracy score, and peer assessment were collected to capture the group and individual diagnostic performances.<br>• A post-survey ranking trainees' preferences of the display systems for performing group medical discussions.	• Diagnostic accuracies were highest when groups used the full-immersive VR compared with the conventional and nonimmersive VR ($\chi 22=9.0$, $p=.01$) displays. Differences between the display systems were more prominent with increasing case complexity ($\chi 22=14.1$, $p<.001$) where full-immersive VR had accuracy scores that were 54.49% and 146.82% higher than conventional and nonimmersive VR, respectively.<br>• The diagnostic accuracies provided by the two cardiologists for each participant did not statistically differ from each other ($t=-1.01$, $p=.31$).<br>• The full-immersive VR was ranked as the most preferred display for performing group CHD discussions by 68% of the participants.	2b

Author (year)	Country	Purpose	Virtual simulator/ platform /system	Study design	Included population	Outcomes measurements	Main findings	Kirkpatrick level
Lowe *et al.*, 2020 [29]	United States	To test the feasibility and acceptability of a 360 VR platform for disaster event decision making for adolescent mass casualty incidents (MCI) explore the feasibility of using a 360 VR experience for the assessment of adolescent disaster readiness and also assess its acceptance among users.	A self-developed 360 VR module simulating an adolescent multi-casualty, mass shooting incident.<br>The 360 VR headset and controls using either an Oculus Go (Face book) or Daydream VR (Google) device.	A cross-sectional observational	Ninety-six (46%) subjects identified as attendings, 66 (32%) as residents, 13 (6%) as medical students, 4 (2%) as emergency medical technicians and 28 (14%) as other.	An anonymous post-simulation 7-question, 5-point Likert survey to describe and rate their experience.	• Based on a 5-point Likert scale, participants felt the 360 VR experience was engaging (median = 5) and enjoyable (median = 5).<br>• Most felt that 360 VR was more immersive than mannequin-based simulation training (median = 5).	1
Orland *et al.*, 2020 [32]	United States	To determine whether VR could improve inexperienced individuals' surgical completion and accuracy of an intramedullary tibial nail procedure, which requires versatile movements that are relevant to the practice of orthopaedic surgery.	The VR platform was from OssoVR (Palo Alto, CA, USA), an orthopaedic VR company that created a simulation of intramedullary tibial nail insertion.	A randomized control trial	Twenty-five first- and second-year medical students were randomly assigned to the technique guide control group (n = 8), the VR group (n = 8), or the VR and technique guide group (n = 9).	• The proportion of participants in each of the three study groups who were able to complete the task.<br>• The proportion of incorrect steps in each group (defined as the number of incorrect steps compared with the number of steps performed).	• A higher proportion of participants in the VR group (6 of 8) and the VR and technique guide group (7 of 9) completed the intramedullary nail than did participants in the technique guide group (2 of 8; $p = 0.01$). There was no difference in completion between the VR groups ($p = 0.89$).<br>• Participants in the VR and VR and technique guide had fewer normalized incorrect steps than did participants in the technique guide group (3.2 ± 0.1 of 16 and 3.1 ± 0.1 of 16 versus 5.7 ± 0.2 of 16, respectively; $p = 0.02$ for comparisons of VR	2b

Author (year)	Country	Purpose	Virtual simulator/ platform /system	Study design	Included population	Outcomes measurements	Main findings	Kirkpatrick level
							groups to technique guide, p = 0.63 between the VR group).	
Zackoff *et al.*, 2020 [34]	United States	To determine whether exposure to an immersive VR curriculum on pediatric respiratory distress improves medical students' recognition of impending respiratory failure.	A self-developed VR environment constructed using the Unity (Unity Technologies, San Francisco, CA) development platform and experienced through an Oculus Rift (Facebook Technolo gies, LLC, Irvine, CA) headset.	A randomized, controlled, prospective study	A total of 168 third-year medical students (78 in intervention).	A free response clinical assessment of video vignettes at the end of the pediatric rotation.	Significant differences between intervention and control were demonstrated for consideration/interpretation of mental status ($p < 0.01$), assignment of the appropriate respiratory status assessment ($p < 0.01$), and recognition of a need for escalation of care ($p = 0.0004$).	2b
Blumstein *et al.*, 2020 [35]	United States	To compare the performance of medical students trained using a VR program to those trained with a standard guide (SG) program in a SawBones simulation of a tibial intramedullary nailing (IMN) procedure	A self-developed SawBones simulation of a tibial IMN procedure run on a standard Osso VR hardware unit utilizing the Oculus Rift VR headset and Oculus Touch motion controllers.	A blinded, randomized prospective study	First and second-year medical students were randomized to SG (n=10) or VR training (n=10) .	Participants were observed performing simulated tibia IMN procedure immediately after training and evaluated by a blinded attending surgeon using procedure-specific checklist and 5-point global assessment scale. Participants returned after 2-weeks for repeat	• Aggregate global assessment scores were significantly higher for VR than SG group (17.5 vs. 7.5, $p < 0.001$), including scores in all individual categories.<br>• The percentage of steps completed correctly was significantly higher in the VR group compared to the SG group (63% vs. 25%, $p < 0.002$).<br>• Average improvement between the first and second phases of the study were higher in the VR group compared to SG group across all 5-categories of the global assessment	2b

Author (year)	Country	Purpose	Virtual simulator/ platform /system	Study design	Included population	Outcomes measurements	Main findings	Kirkpatrick level
		designed to evaluate orthopaedic surgical techniques.				training and evaluation.	scale, and significantly higher for knowledge of instruments (50% vs. 11%, p<0.01).	
Fu *et al.*, 2020 [36]	United States	To evaluate the training effectiveness and participants' learning curves on the virtual basic laparoscopic skill trainer suturing simulator (VBLaST-SS©) and to assess whether the skills were retained after 2 weeks without training.	Virtual basic laparoscopic skill trainer suturing simulator (VBLaST-SS©)	A intervention study	Fourteen medical students from first to third year were randomly assigned to two training groups (7 per group): VBLaST-SS© or FLS, based on the modality of training.	● Participants' performance scores based on the original FLS scoring system.<br>● The cumulative summation (CUSUM) method was used to evaluate learning.	● Six out of seven participants in each group reached the predefined proficiency level after 7 days of training. Participants' performance improved significantly ($p < 0.001$) after training within their assigned group.<br>● The CUSUM learning curve shows that one participant in each group achieved 5% failure rate by the end of the training period.<br>● Twelve out of fourteen participants' CUSUM curves showed a negative trend toward achieving the 5% failure rate after further training.	2b
Lesch *et al.*, 2020 [37]	United States	To examine the TIPS simulation software and evaluate its potential place in the learning environment for surgical trainees..	TIPS (Toolkit for Illustration of Procedures in Surgery) VR software and two 3D haptic drawing pens, which act as facsimiles for laparoscopic instruments.	A crossover study	Second, third, and fourth-year medical students were randomly assigned to perform either a TIPS laparoscopic appendectomy followed by video of a laparoscopic	● A knowledge assessment followed each intervention.<br>● A post-survey used to gather feedback and subjective impressions of the learning experience.	● A 2-tailed Fisher's exact test revealed that the video had greater ease of use ($p = 0.032$), but TIPS had greater utility as a learning tool ($p < 0.001$) and instilled greater confidence in the ability to reproduce procedural steps ($p < 0.001$).<br>● A 2-tailed t test of the average content quiz scores revealed no significant difference in percentage correct between groups on the laparoscopic appendectomy quiz ($p = 0.772$), but a difference favoring video learning on the laparoscopic cholecystectomy quiz (p =	2b

Author (year)	Country	Purpose	Virtual simulator/ platform /system	Study design	Included population	Outcomes measurements	Main findings	Kirkpatrick level
					cholecystectomy (n=19), or video of a laparoscopic appendectomy followed by TIPS laparoscopic cholecystectomy (n=18).		0.042).	
Gurung *et al.*, 2020 [38]	United States	To assess the efficacy of an accelerated proficiency-based training protocol in robotic simulation practice in delivering durable proficiency compared to conventional training methods.	The da Vinci Skills Simulator (dVSS) system	A double-blinded prospective randomized study	16 medical students, in their 3 undergraduate year were randomly assigned equally into either the accelerated skills acquisition protocol (ASAP) or conventional training protocol (CTP).	● The repetitions required to achieve proficiency.<br>● Transfer of the acquired skills to two other non-practiced tasks assessed immediately and prospectively followed through to 3, 6 and 12 months.<br>● Retention of the practiced tasks was assessed along the same timeframe.	● Subjects in the ASAP group acquired proficiency significantly faster in three of the four training tasks: camera control ($p = 0.0002$), suture sponge ($p < 0.0001$), ring walk3 ($p < 0.0001$), and peg board ($p = 0.6936$).<br>● When assessing transfer of skills, there were no significant differences between the two groups: Ring rail 3 ($p = 0.6807$) and Tubes ($p = 0.2240$).<br>● When assessing retention of skills at 3, 6 and 12 months, for all 6 tasks, no significant differences were seen between the ASAP and CTP groups.	2b
Wilson *et al.*, 2020 [45]	United Kingdom	To evaluate the effectiveness of high-fidelity VR and physical model simulation in teaching	Self-developed virtual-reality counterpart of osteotomy models with a	A modified randomised crossover trial	Forty-nine students were randomly allocated to two groups (the VR	Knowledge was assessed by way of questionnaire immediately before, immediately after, and	● In the VR group, participants improved their post-training score by 192.1% ($U=32$; $p<0.00001$).<br>● In the physical models group, participants improved their post-training scores by	2b

Author (year)	Country	Purpose	Virtual simulator/ platform /system	Study design	Included population	Outcomes measurements	Main findings	Kirkpatrick level
		undergraduate orthopaedic concepts.	self-assembled carton headset.		group and the physical models group), with thirty-three finishing the six-week follow-up assessment.	six-weeks after.	163.1% (U=8.5; p<0.00001).<br>● Overall, there was no statistically significant difference in the total means of post-training test scores between the VR and the physical models study groups (U=260.5; p=0.4354).	
Mulligan *et al.*, 2020 [47]	United Kingdom	To establish the construct validity of the ArthroSim VR simulator using validated global rating scales that allow direct comparison with intraoperative performance.	The ArthroSim VR simulator (TolTech Touch of Life Technologies, Aurora, Colorado)	A comparative study	Eight novices (medical students), eight intermediates (registrars), and seven experts (consultants)	● The Imperial Global Arthroscopy Rating Scale (IGARS) and the Arthroscopic Surgical Skills Evaluation Tool (ASSET) scoring systems.<br>● Time to completion.	● The mean IGARS/ASSET scores for the novice, intermediate and expert groups were 14/11, 29/22, and 46/36 respectively. The difference in score between each of the groups was statistically significant (p<0.05).<br>● The average time to completion was 257 seconds, 305 seconds, and 204 seconds respectively. The time to completion was not significantly different between the groups (p=0.6).	2b
Bartlett *et al.*, 2020 [48]	United Kingdom	To investigate how novice performance on a VR hip arthroscopy simulator's visualisation module changed with repeated practice.	Simbionix ArthroMentorVR simulator (3D Systems, Littleton, USA consisted of a computer and monitor, a mannequin, and two haptic feedback devices	A intervention study	Twenty-five medical students	Participants' performance evaluated by the simulator *via* a set of predefined metrics.	● Learning curves were demonstrated by the students, with improvements in time taken, number of collisions (bone and soft-tissue), collision length and efficiency of movement (all p < 0.01).<br>● Improvements in time taken, efficiency of movement and number of collisions with soft-tissue were first seen in session 3 and improvements in all other parameters were seen in session 4. No differences were found	2b

Author (year)	Country	Purpose	Virtual simulator/ platform /system	Study design	Included population	Outcomes measurements	Main findings	Kirkpatrick level
			capable of providing tactile feedback to a pair of instruments via connecting motors				after session 5 for time taken and length of soft-tissue collision. No differences in number of collisions (bone and soft-tissue), length of collisions with bone, and efficiency of movement were found after session 6.	
Lo *et al.*, 2020 [49]	United Kingdom	To provide the first description of the development and utilisation of a virtual 3D flap model in medical education in the undergraduate curriculum.	Autodesk 3DS max (Autodesk, Inc., California, USA) professional modelling software	A intervention study	52 final-year undergraduate	Course evaluation and qualitative feedback	● Student feedback noted an overwhelming preference for the 3D model (74%) as the first choice of educational methodology, versus lectures (26%), textbooks (0%) and research papers (0%) ($p = 0.0035$).<br>● Extraneous cognitive load may be reduced with 3D models, with students rating these as easier to learn from than textbook or research papers ($p = 0.00014$ and $p < 0.00001$, respectively).<br>● No statistically significant difference was found in the perceived ease of learning between 3D models and lectures.	1
Middeke *et al.*, 2020 [51]	Germany	To investigate whether learning outcome elicited by repeated exposure to virtual patient cases is transferable to other cases addressing similar problems.	A self-developed computer-based emergency ward simulation game EMERGE (available for testing at the following Web site: http://57303490.s	A monocentric prospective randomized trial	A total of 69 fifth year medical students were randomized to 3 groups (A, B, AB).Group A was presented with 2 specific cases that were not shown in	An aggregate score reflecting clinical reasoning.	● In the final session, there were no significant between-group differences regarding the sum score reflecting aggregated performance in all 4 cases (A: 66.5 ± 7.2% vs. B: 61.9 ± 12.4% vs. AB: 64.8 ± 11.1%, $p = 0.399$).<br>● An item-by-item analysis revealed that there were no between-group differences regarding correct therapeutic interventions.	2b

Author (year)	Country	Purpose	Virtual simulator/ platform /system	Study design	Included population	Outcomes measurements	Main findings	Kirkpatrick level
			wh.strato-hosting.eu/EMERGE_WebGL_Demo/.)		group B and vice versa. Group AB was exposed to all 4 specific cases.			
Huettl *et al.*, 2020 [56]	Germany	To investigate the influence of laparoscopic camera navigation (LCN) on surgical performance during virtual cholecystectomy (vCHE) using LCN skills (SALAS) score .	A LapSim®, Software Version 2015, produced by Surgical Science (Göteborg, Sweden)	A prospective study	Medical students were divided by the recorded SALAS score into low and medium performers (Group A; 1st-3rd quartile; n = 60) and high performers (Group B, 4th quartile, n = 21).	The numbers of errors regarding centering, horizon adjustment and instrument visualisation as well as manual and verbal corrections by the surgeon were recorded to calculate the SALAS score (range 5-25) to investigate the influence of LCN on surgical performance.	● The SALAS score of the camera assistant correlates positively with the surgeon's overall performance in vCHE ($p < 0.001$), and the surgeon's virtual laparoscopic performance was significantly better in Group B ($p < 0.001$).<br>● A significantly shorter operation time during vCHE was shown for Group B (Median (IQR); Group A: 508 s [429 s; 601 s]; Group B: 422 s [365 s; 493 s];$p = 0.001$).<br>● Frequent gaming and a higher self-confidence to assist during a basic laparoscopic procedure were associated with a higher SALAS score ($p = 0.013$).	2b
Hu *et al.*, 2020 [58]	China	To assess the impact of VR anatomy instruction on the ultrasound competency of novice learners participating in a ultrasonography workshop.	VR software- the Anatomy Master module of MedicalHolodeck (MedicalHolodeck, Switzerland) . VR hardware - VIVE Pro headset (1440x1600	A randomized control study	Third-year medical students were divided into a VR intervention group (n = 47) and a control group (n = 54).	● Participants' competency assessed through ultrasound performance stations on live subjects.<br>● Anatomical and ultrasound image identification ability	● Participants in the intervention group (median = 16; interquartile 13 to 19) had significantly higher scores in ultrasonography task performance tests than the control group (median = 10; interquartile 7 to 14; Mann-Whitney U = 595; $p < 0.01$).<br>● In sub-group analysis, the intervention group performed significantly better in the six out of ten ultrasound tasks.	2b

Author (year)	Country	Purpose	Virtual simulator/ platform /system	Study design	Included population	Outcomes measurements	Main findings	Kirkpatrick level
			resolution, 90Hz refresh rate) with dual controllers (HTC Vive, Taiwan).			measured using multiple choice tests.	● Participants in the intervention group had greater improvement in ultrasonographic image identification multiple choice tests than the control group (Mann-Whitney U = 914; $p < 0.05$).	
Chen *et al*., 2020 [59]	China	To compare the results of the skull virtual learning resource (VLR) with the cadaver skull and a 2D atlas for anatomy education by administering objective questionnaires and perception surveys.	A self-developed coloured and detachable skull VLR being compatible with HTC VIVE CE (High Technology Computer Corporation, Taiwan), a VR headmounted display with a resolution of 2160 × 1200.	A randomized controlled study	A total of 73 Seventy-four clinical undergraduates were conducted with three different groups, namely, the VR group (n = 25), cadaver group (n = 25) and atlas group (n = 23).	Pre- and post-intervention tests comprising a theory test and an identification test. The theory test consisted of 18 multiple-choice questions, and the identification test consisted of 25 fill-in-the-blank questions.	● The participants in all three groups had significantly higher total scores on the post-intervention test than on the pre-intervention test; the post-intervention test score in the VR group was not statistically significantly higher than the post-intervention test score of the other groups (VR: 30 [IQR: 22-33.5], cadaver: 26 [IQR: 20-31.5], atlas: 28[IQR: 20-33]; $p > 0.05$).<br>● The participants in the VR and cadaver groups provided more positive feedback on their learning models than the atlas group (VR: 26 [IQR: 19-30], cadaver: 25 [IQR: 19.5-29.5], atlas: 12 [IQR: 9-20]; $p < 0.001$).	2b

Author (year)	Country	Purpose	Virtual simulator/ platform /system	Study design	Included population	Outcomes measurements	Main findings	Kirkpatrick level
Du *et al.*, 2020 [60]	China	To explore the effect of a multiple-player VR gaming system on anatomy learning.	The HTC Vive system (HTC Corporation, Taiwan) consisting of two controllers, a head-mounted display, and two infrared laser emitter units.	A randomized controlled study	A total of 18 participants were randomly assigned into 3 learning conditions: (1) a textbook reading control group (CG), (2) a single-player VR (SP) group; and (3) a multiple-player VR (MP) group. There were 6 participants in each group.	The scores on the follow-up multiple-choice test, the correct assembly rates, and the motivation inventory scores.	● There was a significant interaction effect of group and timepoint ($p = 0.003$) in the multiple-choice test.<br>● In the CG, the scores on Day 1, Day 5, and Day 12 were significantly different ($p < 0.001$). The scores on Day 5 were significantly higher than those on Day 1 ($p < 0.001$). Although the scores declined slightly on Day 12, they were still significantly higher than those on Day 1 ($p < 0.001$). The SP and MP groups had similar results ($p < 0.001$, $p < 0.001$). The differences between the groups were only significant on Day 12 ($p = 0.003$), not Day 5 ($p = 0.06$). On Day 12, the scores of the MP group were higher than those of the CG ($p = 0.002$).	2b
Chiu *et al.*, 2020 [61]	China	To evaluate the gender differences in the acquisition of robotic suturing skills.	dVSS (Intuitive Surgical Inc., Sunnyvale, CA, USA) for da Vinci version Xi based on Mimic Technologies software .	A comparative study	39 male and 19 female medical students	Instructors assessed participants' robotic suturing and intracorporeal knot-tying performance using an assessment checklist based on the validated the Objective Structured Assessment of Technical Skills	● The metric analysis of the VR task "suture sponge" showed that female students required less time (difference: -170.7 seconds, 95% CI: -247.4 to -94.0) and had fewer errors (error difference: -50, 95% CI: -74.2 to -25.8) to complete the suture sponge exercise compared to male students.<br>● Female students completed more stitches than male students (differences in mean stitch achieved: .35; 95% CI: .06 to .65).	2b

Author (year)	Country	Purpose	Virtual simulator/ platform /system	Study design	Included population	Outcomes measurements	Main findings	Kirkpatrick level
						(OSATS) tool.	● There was no difference in the quality scores of stitches by gender ($p = 0.85$).	
Andersen *et al.*, 2020 [66]	Denmark	To investigate the effects of simulator-integrated tutoring on performance and learning.	The Visible Ear Simulator (version 2.1) , an academic freeware VR temporal bone surgical simulator. A Geomagic Touch haptic device (3D Systems Inc, Rock Hill, SC).	A prospective, educational cohort study	14 medical students in the tutored cohort and 14 in the reference cohort	Final-product performance assessed by 2 blinded raters and simulator-recorded metrics	● Simulator-integrated tutoring had a large and positive effect on the final-product performance while turned on (mean difference = 3.8 points, $p < 0.0001$). However, this did not translate to a better final-product performance in subsequent non-tutored procedures.<br>● The tutored cohort had a better metrics-based score, reflecting higher efficiency of drilling (mean difference = 3.6%, $p = 0.001$).<br>● For the individual metrics, simulator-integrated tutoring had mixed effects both during procedures and on the tutored cohort in general (learning effect).<br>● Simulator-integrated tutoring by green lighting of the volume to be drilled during VR simulation training of mastoidectomy did not induce a better final-product performance but increased efficiency.	2b

**Author (year)**	**Country**	**Purpose**	**Virtual simulator/ platform /system**	**Study design**	**Included population**	**Outcomes measurements**	**Main findings**	**Kirkpatrick level**
Michelet *et al*., 2020 [68]	France	To focus on the impact of computer debriefing on learning acquisition and retention after a screen-based simulation training on neonatal resuscitation designed for midwifery students.	The screen-based digital simulator designed by Medusims—Périna tsims.	A randomized controlled simulation study	A total of 28 fourth-year midw ifery students were randomized 1:1 into the debriefing group and the control group.	● A knowledge questionnaire on neonatal resuscitation.<br>● A self-efficacy rating.<br>● Expert evaluation of technical skills as per the Neonatal Resuscitation Performance Evaluation (NRPE) score and of nontechnical skills as per the Anaesthetists' Non-Technical Skills (ANTS) system	● The participants from the debriefing group reached higher ANTS scores than those from the control group during session 1 (13.25 vs 9; U=47.5; p=.02). Their scores remained higher, without statistical difference during session 2 (10 vs 7.75; p=.08).<br>● The debriefing group had higher self-efficacy ratings at session 2 (3 vs 2; U=52; p=.02).<br>● When comparing the knowledge questionnaires, the significant baseline difference (13 for debriefing group vs 14.5 for control group, p=.05) disappeared at the end of session 1 and in session 2.<br>● No difference was found for the assessment of technical skills between the groups or between sessions.	2b
Fiard *et al*., 2020 [69]	France	To evaluate the ability of students to reproduce the skills acquired on a prostate biopsy simulator in a real-life situation.	The Biopsym simulator, a self-developed simulator for prostate biopsies.	A prospective randomized controlled study	Twenty-two students (arm A< conventional training >(n=10) and arm B<simulator-enh anced training>(n=12).	The transfer of skills was assessed by recording the position of the 12 biopsies performed by each student on an unembalmed human cadaver using a 3D ultrasound mapping device.	● The median score obtained on the simulator at the end of the training was 57% (53-61) for arm A and 66% (59-71) for arm B.<br>● The median score obtained on the cadaver by students trained with the simulator was 75% (60-80), statistically superior to the score obtained by students trained conventionally of 45% (30-60), $p < 0.0001$.<br>● The median score obtained by all students when performing biopsies in a real-life	2b

Author (year)	Country	Purpose	Virtual simulator/ platform /system	Study design	Included population	Outcomes measurements	Main findings	Kirkpatrick level
							situation was 63% (50-80) versus 60% (56-70) for their last training on the simulator.	
Ros *et al.*, 2020 [71]	France	To assess improvement in learning a technique using an immersive tutorial, a 3D video (filmed from the first-person point of view), displayed on a VR application.	The immersive experience was displayed on a Samsung® Gear VR (Samsung Electronics, Seoul, South Korea)	A prospective randomized comparative study	Fourth-year medical students were assigned to Group A (received a technical note, n=88) and Group B (trained using VR, n=85).	A multiple-choice questionnaire completed immediately after the training and again at six months.	The VR group demonstrated significantly better short-term results than the control group (p=0.01). The same trend was seen at six months.	2b
Watari *et al.*, 2020 [73]	Japan	To clarify the effective- ness of Virtual Patient Simulations (VPSs) for improving clinical reasoning skills among undergradu ate medical students, and to compare improvements in knowledge or clinical reasoning skills relevant to specific clinical scenarios.	A VPS program (®Body Interact, Portugal)	A single center pre–post study	Fourth-year medical students (n=169)	A pre-post 20-item multiple-choice questionnaire (10 knowledge and 10 clinical reasoning items) used to evaluate learning outcomes.	Participants showed significant increases in average total post-test scores, both on knowledge items (pre-test: median = 5, mean = 4.78, 95% CI (4.55-5.01); post-test: median = 5, mean = 5.12, 95% CI (4.90-5.43); p-value = 0.003) and clinical reasoning items (pre-test: median = 5, mean = 5.3 95%, CI (4.98-5.58); post-test: median = 8, mean = 7.81, 95% CI (7.57-8.05); p-value < 0.001).	2b
Wada *et al.*, 2020 [74]	Japan	To investigate the usefulness of the 3D imaging technique in	3D reconstruction was performed from pre-operative		Thirty medical students	A multiple-choice questionnaire (MCQ) survey to determine the	• The results of the MCQ showed that most of the medical students were extremely (23%) or very (67%) satisfied with the effect of	1

Author (year)	Country	Purpose	Virtual simulator/ platform /system	Study design	Included population	Outcomes measurements	Main findings	Kirkpatrick level
		laparoscopic transabdominal pre-peritoneal repair (TAPP) as an educational tool for medical students.	computed tomography (CT) data of Six (6) patients who underwent laparoscopic TAPP for inguinal hernia.			level of their satisfaction and understanding of anatomy resulting from the study.	pre-operative 3D simulation on the quality of the surgery.<br>● Most students could understand the surgery anatomy by the 3D simulation extremely well (40%) or very well (47%) and agreed on the usefulness of this procedure for learning anatomy.	
Oussi *et al*., 2020 [78]	Sweden	To assess whether motivation and surgical skill performance differ after basic skills training (BST) using a low-cost (Blackbox) versus laparoscopic high-fidelity simulator(LapMentor) among medical students.	LapMentor (3D Systems Corp) equipped with two mock working handles.	A comparative study	A total of 47 medical students were randomized into two groups that received BST using Blackbox (n = 29) or LapMentor (n = 18).	● Questionnaire regarding expectations of the simulation training.<br>● A visuospatial ability test using the minimally invasive surgical trainer-VR (MIST-VR) simulator.	● Students anticipated mastering LapMentor would be more difficult than Blackbox (p = 0.04).<br>● In those completing the simulation training, a trend toward an increase was noted in how well participants in the Blackbox group liked the simulator training (p = 0.07).<br>● Subgroup analysis of motivation and difficulty in liking the training regardless of simulator was found only in women (Blackbox [p = 0.02]; LapMentor [p = 0.06]).<br>● In the Blackbox group, the perceived difficulty of training, facilitation, and liking the Blackbox training (significant only in women) were significantly correlated with the students' performance in the MIST-simulator.<br>● No such correlations were found in the LapMentor group. in the Blackbox group, the experienced difficulty and facilitation showed an excellent correlation with the actual MIST	2b

Author (year)	Country	Purpose	Virtual simulator/ platform /system	Study design	Included population	Outcomes measurements	Main findings	Kirkpatrick level
							performance, a finding not noted in the LapMentor group.	
Siyar *et al*., 2020 [81]	Canada	To introduce VR simulators as potential tools for the quantitation of physiological tremor in neurosurgery during a tumor resection task.	NeuroTouch platform with haptic feedback utilizing the simulated ultrasonic aspirator held in the dominant hand.	A validation study	A total of 115 subjects including 16 board certified and practicing neurosurgeons, and 7 senior residents post-graduate year (PGY 4-6) who comprised the skilled group (n =23) along with 8 junior residents (PGY 1-3) and 84 medical students who comprised the novices (n= 92).	The means of tremor amplitude obtained from the power spectral density method.	• The skilled group showed less tremor than the novice group in all cases.<br>• The skilled group displayed smaller physiological tremor than the novice group in all cases. In 3 out of 7 cases the difference was statistically significant.	2b
Torda, 2020 [89]	Australia	To evaluate CLASSIE modules which examined student engagement, knowledge gains (self-perceived and measured) and user	A suite of online modules called 'Clinical adaptive student studies in Ethics' or 'CLASSIE' modules Each	A pilot study	Final year medical student (n=52)	• Each tutorial included an identical entry quiz and an identical exit quiz.<br>• Online questionnaire.<br>• Analytics of time	• Engagement and self-perceived knowledge gains were extremely high. Students found these modules realistic, interesting and helpful.<br>• The measured knowledge gains (module exit quiz) were moderate.<br>• User experience was positive overall,	2b

Author (year)	Country	Purpose	Virtual simulator/ platform /system	Study design	Included population	Outcomes measurements	Main findings	Kirkpatrick level
		experience.	consisted of a clinical scenario filmed using VR technology, linked to an adaptive, interactive, online tutorial which explicitly discussed the relevant ethical issues and guidelines.			spent and learning gains extracted from the Smart Sparrow software.	although students were intolerant of any technical glitches.<br>● Student reflections showed high level incorporation of these modules into clinical practice of the students and evidence of knowledge transfer (level 3 Kirkpatrick model of evaluation) in over ¾ of students.	
Liaw *et al.*, 2020 [91]	Singapore	To evaluate a team training program using VR *vs* conventional live simulations on medical and nursing students' communication skill performances and teamwork attitudes.	CREATIVE (Create Real-time Experience and Teamwork in Virtual Environment), where users can create physical and social presences using avatars in a 3D virtual hospital environment.	A randomized controlled trial study	A total of 120 undergraduate medical and nursing students who were rand-omly 1:1 assigned to undertake team training using VR or live simulations.	● Communication performances through team-based simula-tion assessments.<br>● Interprofessional attitude surveys administered before, immediately after, and 2 months after the interventions.	● The team-based simulation assessment revealed no significant differences in the communication performance posttest scores (p=.29) between the virtual and simulation groups.<br>● Both groups reported significant increases in the interprofessional attitudes posttest scores from the baseline scores, with no significant differences found between the groups over the 3 time points.	2b

Author (year)	Country	Purpose	Virtual simulator/ platform /system	Study design	Included population	Outcomes measurements	Main findings	Kirkpatrick level
Jeon *et al.*, 2020 [92]	Korea	To describe Cardiac E-C_Sim, and its use within the Human Physiology course.	A self-developed mathematical computer simulation model of human ventricular myocytes (Cardiac E-C_Sim).	A intervention study	A total of 304 first year medical students (150 students in 2017 and 154 students in 2018).	● Student survey feedback.<br>● Regular tests in each semester of the physiology course.	The survey results and learning outcomes of students suggest the usefulness of Cardiac E-C_Sim to deepen the understanding of heart physiology through active learning rather than didactic lecture-based learning	2b
Jaskiewicz *et al.*, 2020 [101]	Poland	To assess chest compressions (CCs) quality during sudden cardiac arrest (SCA) scenario performed with the use of VR prototype.	Beta version of CPR VR learning software (VR ACT, Octopus VR, Lodz, Poland) implemented for HTC Vive (HTC, Taoyuan, Taiwan) and Noitom Hi5 VR Glove (Noitom International Inc. Miami, Florida, USA).	A cross-sectional design	A total of 91 first-year medical students participated twice in the scenario of SCA - Traditional Scenario (TS) (n=46) and VR Scenario (VRS)(n=45).	● CCs quality was measured with SimPad equipped with Skillreporter software (Laerdal, Stavanger, Norway).<br>● A questionnaire.	● If VRS was the first scenario there were significant differences in CCs depth (VRS - Me = 47 mm [IQR 43 - 52] vs TS - Me = 48 mm [IQR 43 - 55]; $p = .02$) and chest relaxation (VRS - Me = 37% [IQR 5 - 91] vs TS - Me = 97% [IQR 87 - 100]; $p < .001$).<br>● 97.8% of respondents believe that training with the use of VR is more effective than a traditional method ($p < .01$).<br>● Most of the study group (91%, $p < .01$) denied any negative symptoms during the VR scenario.	2b
López Chávez *et al.*, 2020 [102]	Mexico	To explore whether and how different approaches for designing medical educational tools including a 2D version, a gamified	The four versions of MediTool (a non-commercial software) were (i) a 2D desktop version presenting static content;	A comparative case study	A total of 78 undergraduate medical students including 8 first-year students, 6 third-year	● A traditional paper-based exam designed to evaluate the participant's learning performance after studying the clinical cases using a	● The highest average scores on the traditional exams were obtained for the gamified 2D version and the gamified 3D version, whereas the lowest average scores were obtained for the 2D version and the immersive-virtual-reality version.	2b

Author (year)	Country	Purpose	Virtual simulator/ platform /system	Study design	Included population	Outcomes measurements	Main findings	Kirkpatrick level
		2D version, a gamified 3D version, and a gamified immersive-virtual-reality version affect students' learning performance.	(ii) a gamified 2D version including badges, challenges, and other gamify-cation elements; (iii) a gamified 3D version using 3D avatars and non-immersive virtual reality; (iv) a gamified immersive virtual reality version.		students, 31 fourth-year students, and 33 fifth-year students.	particular version of MediTool.<br>● A 12-item Likert-type questionnaire based on the Technology Acceptance Model (TAM) to evaluate the applications regarding usefulness, usability, and gamification.	● Based on the participants' perception, the gamification of the immersive-virtual-reality version helped the most to improve their learning performance in comparison with the gamified 2D and 3D versions.	
Alharbi *et al.*, 2020 [104]	Saudi Arabia	To determine the effectiveness of 3D-VR in knowledge retention in human anatomy courses as compared to traditional teaching methods among medical students.	A self-developed 3D-VR human anatomy models installed on iPads	A convergent mixed methods design	A total of 178 third-year medical students includin g 104 males and 66 females were divided into the control group (n=89) and the experimental group (n=89).	● Pre- and post-tests administered through the objective structured practical examination (OSPE).<br>● The group discussions.	● Male students who used the 3D-VR tool had significantly higher short- and long-term knowledge scores than males who used the traditional methods. Meanwhile, females who used traditional methods showed significantly higher short-term knowledge scores than females who used 3D-VR.<br>● Medical students described 3D-VR as a learning tool, with a great deal to offer in learning human anatomy as compared to traditional methods.	2b

Table S2. Summary of study characteristics and main findings of included studies published in 2021(n=50).

Author (year)	Country	Purpose	Virtual simulator/ platform /system	Study design	Included population	Outcomes measurements	Main findings	Kirkpatrick level
Hu *et al.*, 2021 [10]	China	To design a serious computer game to train undergraduate medical students in neonatal resuscitation in a cost-friendly and accessible way and to examine whether serious game-based training improves long-term knowledge retention in medical students.	NEOGAMES (Neonatal Resuscitation Simulation Game Designed for Medical Students) on the National Virtual Simulation Experiment Teaching Project Sharing Platform (http://www.ilab-x.com/[in Chinese])	A controlled intervention Study	Eighty-one undergraduate medical students (41 and 40 participants in the game and control groups, respectively)	Three written tests, pre- and post-training knowledge tests and a follow-up test after 6 months	● Significant short-term knowledge improvement was noticed only for male students in the game group based on their 5.2-point higher test scores than those of the controls ($p = 0.006$).<br>● Long-term knowledge improvement at 6 months was identified for both male and female students in the game group, with test scores 21.8 and 20 points higher, respectively, than those of the controls ($p < 0.001$). The long-term knowledge retention in the game group was almost 3 times higher than that in the control group.	2b
Kenngott *et al.*, 2021 [50]	Germany	To evaluate among different healthcare professions the potential of an interactive and immersive VR environment for liver surgery that integrates all relevant patient data from different sources needed for planning and training of procedures.	An interactive and immersive VR system (IMHOTEP) for visualizing surgical planning data with a 3D VR framework together with all other clinical information using the comer-cially available head-mounted display (HMD) Oculus Rift™.	A comparative study	A total of 158 participants (57 medical students, 35 resident surgeons, 13 attending surgeons and 53 nurses)	Standardized questionnaire	● 89.9% found the VR system agreeable to work with.<br>● Participants generally agreed that complex cases in particular could be assessed better (94.3%) and faster (84.8%) with VR than with traditional 2D display methods.<br>● The highest potential was seen in student training (87.3%), resident training (84.6%), and clinical routine use (80.3%). Least potential was seen in nursing training (54.8%).	1

Author (year)	Country	Purpose	Virtual simulator/ platform /system	Study design	Included population	Outcomes measurements	Main findings	Kirkpatrick level
Morgan *et al.*, 2021 [41]	United Kingdom	To explore the effectiveness of VS to sustain medical education for various medical specialties during the COVID-19 pandemic.	Simulation via Instant Messaging – Birmingham Advance (SIMBA), a self-developed moderator through WhatsApp®.	A intervention study	A total of 204 participants consisted of consultants (n = 24), specialty trainees (n = 138), core medical or internal medicine trainees (n = 10), foundation doctors (n = 7) and medical students (n = 14).	● Self-reported confidence levels were obtained from pre- and post-SIMBA surveys.<br>● Perceptions towards SIMBA's impact on their clinical behaviour.	● Significant improvement was observed in participants' self-reported confidence (overall n = 204, p<0.001).<br>● Participants reported improvements in clinical competencies: patient care 52.0% (n = 106/204), professionalism 30.9% (n = 63/204), knowledge on patient management 84.8% (n = 173/204), systems-based practice 48.0% (n = 98/204), practice-based learning 69.6% (n = 142/204) and communication skills 25.5% (n = 52/204).<br>● Participants felt the sessions were relevant, engaging, and accommodated their learning styles.	2b
Liaw *et al.*, 2021 [90]	Singapore	To explore the experiences of students and facilitators on the use of three-dimensional virtual world (3DVW) for interprofessional team-based virtual simulation.	A self-developed Create Real-time Experience And Teamwork In Virtual Environment (CREATIVE) software using the Unity 5 games engine (Unity Technologies, San Francisco, CA).	A qualitative descriptive study	30 full-time senior students from six healthcare courses including medicine (n = 5), nursing (n = 5), pharmacy (n = 4),physiotherapy (n = 5), occupational therapy (n = 6) and medical social work (n = 5).	Thematic analysis based on focus group discussions attended by 30 students and individual interviews attended by 12 facilitators	● Four themes emerged from their experiences: the "wow experience", authentic experience on collaborative care, ease of learning, and preeminent role of the facilitator.<br>● The simulation provided the "wow" experiences through contextual, collaborative and experiential learning approaches.<br>● Despite technical challenges, the participants were wooed by the comforts of learning from home and the psychological safety in virtual environment.<br>● The facilitators played a critical role in optimizing learning engagement to win	1

Author (year)	Country	Purpose	Virtual simulator/ platform /system	Study design	Included population	Outcomes measurements	Main findings	Kirkpatrick level
							learners over.	
Dekhtyar *et al.*, 2021 [15]	United States	To test the hypothesis that the Symptom to Diagnosis (S2D) diagnostic reasoning approach videos paired with practice virtual patient encounter simulations (VPES) could improve the diagnostic accuracy in medical students as evidenced by their ability to diagnose new simulated cases with diagnoses not previously encountered.	i-Human Patients (IHP) software (Kaplan Company, Sunnyvale, CA,USA)	A multi-institutional randomized control trial	A total of 285 medical students were randomized to receive training in either abdominal pain (AP) (n=141) or loss of consciousness (LOC) (n=144).	Diagnostic accuracy was assessed by completing a multiple-choice question (MCQ) examination and virtual patient encounters, and compared between baseline and post intervention. The AP group served as a control for the LOC group and vice versa.	● Compared with baseline, both the AP and LOC groups had marked post-intervention improvements in obtaining a correct final diagnosis; a 27% absolute improvement in the AP group ($p<0.001$) and a 32% absolute improvement in the LOC group ($p<0.001$).<br>● Compared with controls (the groups trained in the alternate symptoms), the rate of correct diagnoses increased by 13% but was not statistically significant ($p=0.132$).<br>● Compared with controls, the completeness and efficiency of the differential diagnoses increased by 16% ($\beta=0.37$, $p<0.001$) and 17% respectively ($\beta=0.45$, $p<0.001$).	2b
Austin *et al.*, 2021 [16]	United States	To develop a hybrid simulation model that accommodated a reduced number of learners in simulation center to allow for physical distancing, seamlessly integrated with an online	● Adult simulations: SimMan 3G (Laerdal Medical, Stavanger, Norway) high-fidelity manikin with vital signs displayed in the simulation room	A intervention study	Fourteen residents participated in live simulation; six residents and six medical students comprised the remote group. After intervention,	An electronic survey was then sent to remote participants to rate the effectiveness of the overall virtual	● For the adult simulation, 100% of respondents felt that the virtual experience was either better (n = 6) or neutral (n = 4) to a traditional live simulation.<br>● For the pediatric simulation, 92% of respondents felt that the virtual experience was either better (n = 2) or neutral (n = 9) to a traditional live simulation, suggesting high	1

Author (year)	Country	Purpose	Virtual simulator/ platform /system	Study design	Included population	Outcomes measurements	Main findings	Kirkpatrick level
		integrated experience for remote learners.	on an in-room display.<br>● Pediatric simulations: Pediatric HAL (Gaumard Scientific, Miami, FL) with vital signs displayed on an in-room monitor.		23 survey responses were collected: 52.2% (12) from the live session and 47.2% (11) from the virtual session.	simulation experience compared to a live, in-person simulation (three-point Likert scale, better to worse).	rates of satisfaction for the online observation and debriefing sessions.	
Foohey *et al.*, 2021 [79]	Canada	To describe the Virtual Resus Room (VRR)—a free, novel, open access resource for running collaborative online simulations.	Virtual Resus Room (VRR) https://virtualresusroom.com/.	A intervention study	Forty-six students and 11 facilitators	● Students and facilitators completed a post-session survey to assess usability and acceptability, applicability for learning or teaching, and fidelity.<br>● Students took a knowledge test pre- and post-session.	● Facilitators and students rated the VRR's usability and acceptability, applicability for learning and teaching, and fidelity highly.<br>● Students showed a significant improvement in their post-session (mean = 89.06, SD = 9.56) compared to their pre-session knowledge scores (mean = 71.17, SD = 15.77; $t(34) = 7.28$, $p < .001$, with a large effect size Cohen's $d = 1.23$).<br>● Two perceived learning outcomes were identified: content learning and communication skills development.<br>● The total time spent (in minutes) facilitating VRR simulations (mean = 119, SD = 36) was significantly lower than time spent leading in-person simulations (mean = 181, SD = 58; $U = 20.50$, $p < .008$).	2b

**Author (year)**	**Country**	**Purpose**	**Virtual simulator/ platform /system**	**Study design**	**Included population**	**Outcomes measurements**	**Main findings**	**Kirkpatrick level**
Yang *et al.*, 2021 [17]	United States	To describe the rapid creation and implementation of a fully online simulation-based pediatric emergency medicine training intervention for medical student learners using existing simulation center staff (faculty, technicians, actors) and resources (simulation technology, scenario files), and report student and faculty feedback on the intervention.	A self-developed remote desktop software was used to access the centers Laerdal LLEAP Software (Laerdal Medical, Stavanger, Norway) and preprogrammed scenario files.	A intervention study	All 48 students and 10 faculty completed the pediatric scenarios	Feedback on the simulation session was collected via an online survey including 6 statements using 5-point Likert scales and 2 demographic questions from faculty and students at the end of each session.	● Ninety percent of the students agreed with the statements, "*I am more comfortable with pediatrics after this session*," "*participating improved my pediatric knowledge/skills*," "*this session was more useful than other learning activities I am involved in at this time*."<br>● Seventy percent of the students agreed with the statement, "*I learned as much from observing as when I was actively involved*."<br>● All faculty agreed with the statement, "*this was an effective educational strategy compared to other distance learning*."<br>● Most faculty (60%) disagreed with the statement, "*virtual simulation was equal to or superior to in-person simulation*."<br>● All students and faculty strongly agreed with the statement, "*I would highly recommend this to others*."	1
Putnam *et al.*, 2021 [18]	United States	To assess usability and feasibility of combined interactive instructional videos and a novel VR trainer for healthcare professionals and to evaluate the impact of this combination on	VR trainer using HoloLens® technolog y (Microsoft Corp., Redmond, WA) simulating a pediatric critical airway event.	A intervention Study	Forty-one participants including 8 4th-year medical students, 6 residents, 3 fellow, 10 nurse and 14 faculty.	● Pre-test and post-test of pediatric airway emergency management.<br>● A survey of their perceptions	● Overall, both interventions were well received. Positive perceptions included realism, interactivity, and active learning environment. Negative comments focused on video speed and the VR trainer learning curve.<br>● Participants reported preferences for future training of pediatric airway events to include videos and VR trainers, with or without	2b

Author (year)	Country	Purpose	Virtual simulator/ platform /system	Study design	Included population	Outcomes measurements	Main findings	Kirkpatrick level
		learners' knowledge of critical airway events in children.				of the videos and VR trainer	didactic lectures.<br>● Most areas of knowledge showed slight to significant improvements following the interventions. Specifically, questions on pediatric anatomy, anaphylaxis, Heimlich maneuver, and foreign body removal showed the highest improvement in scores ($p < 0.05$).	
Bogomolova *et al.*, 2021 [82]	The Nether-lands	To describe the development of a virtual 3D anatomy assessment and the perspectives from teachers and students on the use of this assessment tool in a medical curriculum.	An AR application for HoloLens®, Version 1, (Microsoft, Corp., Redmond, WA) was developed to integrate the anatomy test and anatomical 3D model into a virtual 3D assessment scenario.	A design-based research	After first redesign, the virtual 3D assessment scenario was tested by six undergraduate medical students. In the final iterative cycle, the assessment scenario was evaluated by four students and six teachers.	Feedback regarding personal experience and practical use was collected through a standardized self-reported questionnaire.	Virtual 3D assessment can address several challenging aspects of the growing misalignment between learning, assessment, and clinical practice.	1
Plackett *et al.*, 2021 [43]	United Kingdom	To identify the data gathering patterns of final year medical students while using electronic Clinical Reasoning Educational Simulation Tool (eCREST) and how	The electronic Clinical Reasoning Educational Simulation Tool (eCREST)	A mixed methods study	A total of 148 students (78 in the intervention group and 70 in the control group) from 3 medical schools.	● A trial of eCREST for measuring Clinical reasoning skills outcomes.<br>● Qualitative think-aloud	● Qualitative data showed students reported that eCREST promoted thoroughness by prompting them to continuously reflect and allowing them to practice managing uncertainty.<br>● The trial results indicated that students in the intervention group were more thorough than those in the control groups when gathering data.	2b

Author (year)	Country	Purpose	Virtual simulator/ platform /system	Study design	Included population	Outcomes measurements	Main findings	Kirkpatrick level
		eCREST influences the patterns.				and semi-structured interview.	● Reanalysis of the trial data revealed that the intervention group was significantly more likely to display a Thorough data gathering pattern than controls (21/78, 27% vs 6/70, 9%) and less likely to display a Succinct pattern (13/78, 17% vs 20/70, 29%; χ^2_3=9.9; p =.02). Other patterns were similar across groups.	
Rudolph i-Solero *et al.*, 2021 [85]	Spain	To assess student perception, impact on learning, and eventual correlation of game results with post-exposure tests and course grades.	A competition-based learning game about radiological anatomy and signs named The League of Rays developed in Second Life.	A intervention study	Fifty-two third-year undergraduate medical students	● A cognitive load test.<br>● A questionnaire about the experience and a post-exposure knowledge test.	● Students were assessed different aspects of the experience with a mean score ⩾ 7.8 on a 10-point scale, highlighting the participation of the teacher (9.3 ± 1.1), the educational contents (8.8 ± 1.4) and the usefulness for their education (8.7 ± 1.4).<br>● Participants obtained better post-exposure test results ($p < 0.007$) and better course grades ($p < 0.021$) than non-participants did.	2b
Rudolph i-Solero *et al.*, 2021 [86]	Spain	To reproduce a competitive online game based on self-guided presentations and multiple-choice tests in a mandatory format, to evaluate its development and	A competition-based learning game about radiological anatomy and signs named The League of Rays developed in Second Life.	A comparative study	In 2016 and 2017, respectively, 191 and 182 third-year medical students participated in the game as a mandatory course activity.<br>In 2015, 90	● Perception questionnaire to be answered using a five-point Likert scale.<br>● Eight general	● The mean (±SD) score of the game was 74.7% (±19.5%) in 2015, 71.2% (±21.5%) in 2016 and 67.5% (±21.5%) in 2017 ($p < 0.01$).<br>● Participants valued positively the organization and educational contents but found the virtual world less attractive and the game less interesting than in the voluntary edition.<br>● The experience globally was rated with	2b

Author (year)	Country	Purpose	Virtual simulator/ platform /system	Study design	Included population	Outcomes measurements	Main findings	Kirkpatrick level
		student perceptions compared to a voluntary edition.			third-year medical students participated in the voluntary edition.	aspects of the project to be evaluated on a scale of 1-10 points.<br>● The grades from the final course examination.	8.2 (±1.5), 7.8 (±1.5) and 7.1 (±1.7) mean points (±SD) in a ten-point scale, in the 2015, 2016 and 2017 editions respectively ($p < 0.05$).	
Shu *et al.*, 2021 [20]	United States	To integrate Weasis into a web-based reporting system in order to establish a freeware, platform-independent, image database simulating a real-world Picture Archiving and Commu- nication System(PACS).	A self-developed open-source dcm4chee-based PACS named "Weasis" .	A brief survey	A total of 20 medical students and 20 residents/fellows	An online survey	● Most medical students wanted more hands-on (i.e., PACS) experience.<br>● Residents/fellows liked Weasis and think it improves their image interpretation skills<br>● Neither medical students nor residents thought it was difficult to navigate Weasis.<br>● Both medical students and residents wanted Weasis to be incorporated into the curriculum.	1
Greuter *et al.*, 2021 [22]	United States	To compare the spatial orientation between traditional 2D images and 3D VR models in neurosurgical residents or medical students.	SpectoVR software based on patient-specific DICOM data.	A prospective randomized study	A total of 80 participants including 40 4th-6th year medical students and 40 residents.	An online questionnaire assessing the time to aneurysm detection as well as a spatial anatomical	● Overall, the time to aneurysm detection was shorter in the 3D VR model compared to 2D images, with a trend toward statistical significance (25.77±37.26 vs 45.70±51.94 seconds, $p = 0.052$).<br>● No significant difference was observed for residents (3D VR 24.47±40.16 vs 2D 33.52±56.06 seconds, $p = 0.564$), while in	2b

Author (year)	Country	Purpose	Virtual simulator/ platform /system	Study design	Included population	Outcomes measurements	Main findings	Kirkpatrick level
						description.	students a significantly shorter time to aneurysm detection was measured using 3D VR models (26.95±35.39 vs 59.16±44.60 seconds, p = 0.015).<br>● No significant differences between the modalities for anatomical and descriptive spatial mistakes were observed.<br>● Most participants (90%) preferred the 3D VR models for aneurysm detection and description, and only 1 participant (5%) described VR-related side effects such as dizziness or nausea.	
Elzie & Shaia, 2021 [24]	United States	To use VR to allow students to "become a patient" virtually embodying their daily activities, symptoms, and interactions with caregivers.	Embodied Labs software with Oculus Rift goggles	A pilot study	A total of 145 first-year medical students	Post-survey	● Data generated through surveys and reflections indicated a high level of place illusion, plausibility, and embodiment of users. This high level of immersion generated an increase in comfortability with talking about end-of-life issues, produced a better understanding of what patients and their families experience, and promoted a change in the way students would approach clinical skills.<br>● Analysis of reflections indicated a high level of empathy for the patient and his family members.<br>● Overall, the activity was highly received by students as a valuable learning activity.	1

Author (year)	Country	Purpose	Virtual simulator/ platform /system	Study design	Included population	Outcomes measurements	Main findings	Kirkpatrick level
Bishop *et al.*, 2021 [26]	United States	To further evaluate the construct validity and interobserver reliability of a hip arthroscopy virtual simulator using the Arthroscopic Surgery Skill Evaluation Tool (ASSET) global rating scale.	A diagnostic arthroscopy and a loose body retrieval simulation on the VirtaMed Arthros Hip Simulator (Zurich, Switzerland)	A validation Study	Thirty participants including 12 novices (8 medical students, 4 postgraduate year [PGY] 1-2), 5 intermediate trainees (PGY3-4), 9 senior trainees (PGY5 and fellows), and 4 attending faculty	• The ASSET global rating scale.<br>• Individual simulator metrics were recorded and then compiled to generate a total simulator score (TSS).	• Prior simulation experience ($p \leq 0.01$) correlated with higher TSS and higher ASSET, while video game experience correlated with higher TSS on the diagnostic module only ($p = 0.004$).<br>• There was a significant difference in ASSET score among all experience groups ($p < 0.04$). Novices had the lowest mean ASSET whereas experts had the highest mean ASSET with a difference of 17.4 points.<br>• Overall performance on the surgical module significantly correlated with the ASSET score ($r = 0.444$, $p = 0.016$). There was a significant positive correlation among higher ASSET and number of loose bodies retrieved, operation time, camera path and grasper path length, and percentage of cartilage injury.	2b
Atli *et al.*, 2021 [28]	United States	To assess the effects of a year-long medical school preclinical elective course using a comprehensive, multi-component teaching model with an interactive and immersive VR system as a primary mode of	The self-developed Surgical Theater Precision VR system consisting of a VR headset, 1 controller, the computer monitor, and the surgical theater software.	A intervention study	Twelve second-year medical students	• The pre-course and post-course competency confidence surveys.<br>• The post-course VR experi-	• At course conclusion, 100% of students reported significantly higher competency confidence levels on all topics, and 100% agreed utilizing VR helped them gain a deeper understanding of neuroanatomy/neurosurgery.<br>• 92% agreed that using VR helped them better retain the anatomical/functional details of the brain/spine, and 69% better understand neurosurgical skills taught, respectively.	1

Author (year)	Country	Purpose	Virtual simulator/ platform /system	Study design	Included population	Outcomes measurements	Main findings	Kirkpatrick level
		teaching neurosurgical procedures, pathologies, and neuroanatomy.				ence survey	● 100% of students found the course to be a valuable learning experience and VR a useful learning tool.	
Zackoff *et al.*, 2021 [30]	United States	To establish competency standards related to student recognition of impending respiratory failure using an immersive VR platform, using the clinical scenario of an infant admitted with bronchiolitis.	A self-developed OculusRift VR platform.	A cross-sectional observational study	Twenty-six fourth year medical students	Video recordings were rated by 2 pair of physician reviewers blinded to student identity.	● Student performance of 8 observable behaviors was found to be most predictive of a rating of competent, with a 91% probability.<br>● Correctly stating that the patient required an escalation of care had the largest contribution toward predicting a rating of competent, followed by commenting on the patient's increased heart rate, low oxygen saturation, increased respiratory rate, and stating that the patient was in respiratory distress.	2b
Melnyk *et al.*, 2021 [31]	United States	To evaluate the impact of gaze-augmented training for robotic surgical training in comparison with traditional, movement-based training in robotic surgery-naïve participants.	The VR-based da Vinci Skills Simulator (DVSS) (Intuitive Surgical, Sunnyvale, CA) with Head-Mounted Eye-Trackers System (Pupil Labs, Berlin, DE) fit.	A randomized controlled trial	Medical students were randomized into gaze training (GT, n = 9) and movement training (MT, n = 8) groups.	● DVSS scores during training and multitasking.<br>● Metrics collected from the HMET (gaze patterns and gaze entropy).	● Total score, efficiency, and penalties improved significantly over the training in both groups; the GT group achieved higher scores on every attempt.<br>● Total scores in the GT group were higher than the MT group postvideo review (20.3 ± 21.8 vs 3.0 ± 6.2, p = 0.047), after coaching repetitions (61.8 ± 18.8 vs 30.1 ± 26.2, p = 0.01), and at the last training attempt (73.0 ± 16.5 vs 63.1 ± 17.4, p = 0.247).<br>● During multitasking, the GT group maintained higher total scores (75 ±	2b

Author (year)	Country	Purpose	Virtual simulator/ platform /system	Study design	Included population	Outcomes measurements	Main findings	Kirkpatrick level
							10.1 vs 63.3 ± 15.3, p = 0.01), efficiency (86.3 ± 7.4 vs 77.4 ± 11.2, p = 0.009), and superior secondary task performance (error: 6.3% ±0.06 vs 10.7% ± 0.11, p = 0.20).<br>● Gaze entropy (cognitive-load indicator) and gaze pattern analysis showed similar trends.	
Wang *et al.*, 2021 [33]	United States	To determine the impact of simulator practice on task completion time, radiation use, success rate, and overall quality in a simulation of placing a distal locking screw.	The TraumaVision VR Simulator (Swemac, Sweden)	A prospective, randomized control trial	Twenty-eight volunteer novice trainees (medical and premedical students) were divided into the simulator group and the control group.	Pretest and posttest on the simulator. The primary outcome variables were simulator-collected task completion time, success rate, radiation exposure time, and overall score.	● No notable difference existed between groups for pretest completion time, radiation use, success rate, or overall score.<br>● No notable difference in posttest radiation use or overall procedure score was found between groups.<br>● A significant difference existed in posttest total completion time (trained = 251.2 ± 103.4; control = 497.3 ± 223.1; p = 0.001) and success rate (64.3% versus 100%; p = 0.041) between groups. A significant difference existed in variance between groups for completion time (p = 0.029).	2b
Young *et al.*, 2021 [39]	United States	To assess the use of VR delivered remotely via video teleconferencing for addressing a key experience gap while navigating coronavirus disease	A 1.5-hour video teleconferencing session with a physician facilitator donning a VR headset and screen sharing interactive VR cases of a hospitalized	A prospective pilot study	A total of 140 third-year medical students on their pediatric clerkship	Surveys	● A majority of students reported VR captured their attention (78%) with minimal side effects.<br>● Students reported remote VR training as more effective (p < .001) than reading and online learning and equally or more effective (p < .001) than didactic teaching.	1

Author (year)	Country	Purpose	Virtual simulator/ platform /system	Study design	Included population	Outcomes measurements	Main findings	Kirkpatrick level
		2019 limitations on training.	infant with respiratory distress.				• Most students (80%) rated remote VR as less effective than bedside teaching.	
Chaffkin *et al.*, 2021 [40]	United States	To evaluate the feasibility of a VS format to prepare medical students for collaborative participation in managing agitation alongside house staff.	The virtual session on agitation management to fit within a two-hour block in the psychiatry clerkship Curriculum occurred using Zoom Videoconferencing technology (Zoom Video Communications, San Jose, CA).	A pilot intervention Study	A total of 43 medical students	Pre- and post-test assessments	• Student perception of the importance of attending to safety did not appear to change between the pre-survey and the post-survey (from 4.5±0.74 to 4.7±0.71, p=0.279).<br>• The recognition of managing agitation as an important skill increased from 4.5±0.50 to 4.9±0.35, p<0.001.<br>• Students' confidence in helping manage agitation increased from 2.2±0.75 to 3.2±00.75, p<0.001.<br>• Students' worries about working with psychiatric patients decreased from 2.7±0.87 to 2.2±0.91, p<0.001.	2b
Pickering *et al.*, 2021 [44]	United Kingdom	To explore the impact of a mixed reality (MR) application focused on long spinal cord sensory and motor pathways in comparison to an existing resource already embedded within an active curriculum (e.g., anatomy drawing screencasts).	• Students were exposed to the MR application via the room's projector that was physically-tethered to a Lenovo ThinkPad X1 Carbon with the processor i7-8550U, memory 16GB DDR3 and with solid state drive 512GB (Lenovo, Quarry Bay, Hong Kong).	A quasi-randomized control trial	A total of 200 Year 2 medical students were divided into the Screencast and MR Application Groups (n=127 and 78, respectively)	Pre- and post-test	• Similar patterns of learner gain were observed between the two groups.<br>• Only the multiple-choice questionnaires (MCQs) were shown to be answered significantly higher with the screencast group.	2b

Author (year)	Country	Purpose	Virtual simulator/ platform /system	Study design	Included population	Outcomes measurements	Main findings	Kirkpatrick level
			● The laptop was remotely tethered to the Microsoft Holo-Lens 1 development edition (Windows 10 SDK build version 1809, codenamed "Redstone 5") .<br>● Head-set (Microsoft Corp., Redmond, WA)					
Fukuta *et al.*, 2021 [46]	United Kingdom	To gain an insight into students' receptiveness to the use of 360° video for a virtual operating theatre orientation and its potential use.	A self-developed "virtual" 360° video orientation generated by utilizing a 360° camera. A head mount holding specific camera onto the head of student actor.	A intervention study	Thirty-four second-year medical students	● Knowledge gain measured with use of a questionnaire.<br>● Self-reported confidence using a 7-point Likert scale	The students' knowledge improved from 38.4% to 78.2% ($p < 0.01$) as well as self-reported confidence from 4.3 to 6.1 ($p < 0.01$).	2b
Huettl *et al.*, 2021 [52]	Germany	To compare 3D PDFs, 3D printed models (PR) and VR 3D models with regard to anatomical orientation and personal preferences in a high volume liver surgery	The game engine Unity (version 2019.2.14f1, Unity Technologies, San Francisco, California, USA) with VR headsets.	A comparative study	Thirty participants including 10 medical students, 10 residents, 5 fellows and 5 hepatopancreatobiliary (HPB) experts	● Time needed to specify the tumor location.<br>● A score factoring in correct,	● Participants named significantly more correct segments in VR ($p=0.040$) or PR ($p=0.036$) compared to PDF.<br>● Tumor assignment was significantly shorter with 3D PR models compared to 3D PDF ($p<0.001$) or VR application ($p<0.001$).<br>● Regardless of the modality, HPB experts were significantly faster (24±8 vs. 35±11 sec;	2b

Author (year)	Country	Purpose	Virtual simulator/ platform /system	Study design	Included population	Outcomes measurements	Main findings	Kirkpatrick level
		center.				wrong and missing segment assignments.<br>● Standardized test/ questionaires for spatial thinking and seeing, vegetative side effects and usability.	p=0.014) and more often correct (0.87±0.12 vs. 0.83±0.15; p<0.001) than medical students.<br>● Test results for spatial thinking and seeing had no influence on time but on correctness of tumor assignment.<br>● Regarding usability and user experience the VR application achieved the highest scores without causing significant vegetative symptoms and was also the most preferred method (n=22, 73.3%) because of the multiple functions like scaling and change of transparency.<br>● Ninety percent (n=27) stated that this application can positively influence the operation planning.	
Albrecht *et al.*, 2021 [53]	Germany	To assess the face, content, and construct validity of the novel VR otoscopy simulator and the applicability to otologic training.	A commercially available high-fidelity VR otoscopy simulator (Earsi Otoscope; VRMagic).	Part 1:a cross-sectional study.<br><br>Part 2: a prospectively designed controlled trial	Part 1: 39 medical students and 25 otolaryngologists<br>Part 2: 51 medical students and 25 otolaryngologists	● Face and content validity assessed with a questionnaire using a 6-point Likert scale.<br>● A modified	● The ratings suggested good face and content validity as well as usefulness and functionality of the simulator.<br>● The otolaryngologists significantly outperformed the student group in all categories measured (p < .0001), suggesting construct validity of the simulator.	2b

Author (year)	Country	Purpose	Virtual simulator/ platform /system	Study design	Included population	Outcomes measurements	Main findings	Kirkpatrick level
						objective Structured Assessment of Technical Skills.<br>● Time to complete the task and the percentage of the assessed eardrum surface.		
Klemm *et al.*, 2021 [54]	Germany	To evaluate the feasibility of a VR app to educate health care professionals and medical students about Inflammatory arthritides (IA).	An educational VR app (called Rheumality) equipped with VR set (VR Headset HTC VIVE), motion controller, and Lighthouse Tracking.	A intervention Study	A total of 125 participants including 66 medical students, 50 physicians,5 researchers, and 4 other health care professionals.	An online questionnaire	● The participants rated the VR app as excellent, with a mean rating of 9.0 (SD 1.2) out of 10, and many participants would recommend use of the app, with a mean recommendation score of 3.2 (SD 1.1) out of 4.<br>● A large majority (120/125, 96.0%) stated that the presentation of pathological bone formation improves understanding of the disease.	1
Issleib *et al.*, 2021 [55]	Germany	To compare a conventional cardiopulmonary resuscitation (CPR) training with a VR training aiming to	Leardal® QCPR Mannequin is connected to the VR system which was developed in cooperation	A controlled randomized study	Fifty-six undergraduate first year medical students in the intervention group and 104 participants	● No flow time in a final 3-min Basic Life Support (BLS) examination.	● The no flow time was significantly shorter in the control group (Mean values: control group 82 s vs. intervention group 93 s; p = 0.000).<br>● In the CSA participants of the intervention group had a higher learning gain in 6 out of	2b

Author (year)	Country	Purpose	Virtual simulator/ platform /system	Study design	Included population	Outcomes measurements	Main findings	Kirkpatrick level
		demonstrate: (a) non-inferiority of the VR intervention in respect of no flow time and (b) superiority in respect of subjective learning gain.	between Universitätsklinikum Hamburg Eppendorf and VIRE ED.		to the control group.	• Learning gain assessed with a comparative self-assessment (CSA) using a questionnaire at the beginning and the end of the course.	11 items of the questionnaire ($p < 0.05$).	
Mok *et al.*, 2021 [57]	China	To promote a new approach consisting of a shared and independent study platform for medical orthopedic students, to compare traditional tendon repair training with VR simulation of tendon repair, and to evaluate future applications of VR simulation in the academic medical field.	All VR simulators were classified as HTC Vive VR, and the software was SteamVR (JinKe Lu)	A parallel-design randomized controlled trial	Senior medical students were randomly allocated to VR (n=61) or control (n=60) groups.	The final assessment for the medical students involved performing tendon repair with the "Kessler tendon repair with 2 interrupted tendon repair knots" (KS) method and the "Bunnell tendon repair with figure 8	The overall performance (a total score of 35) of the VR group using the KS method and the BS method was significantly higher ($p <.001$) than that of the control group. Participants who received VR simulator training had a significantly higher score on the global rating scale than those who received traditional tendon repair training ($p<.001$).	2b

Author (year)	Country	Purpose	Virtual simulator/ platform /system	Study design	Included population	Outcomes measurements	Main findings	Kirkpatrick level
						tendon repair" (BS) method on a synthetic model. The operative performance was evaluated using the global rating scale.		
Rosenfeldt Nielsen *et al.*, 2021 [62]	Denmark	To evaluate the effect of immersive VR learning for training medical students in basic clinical ultrasound, and to explore if VR learning had an effect on hand-eye coordination skills and if the medical students wanted more VR learning.	A Samsung Galaxy Tab A (Samsung Group, Seoul, South Korea) was used for e-learning.<br>A Pico Neo (Pico Interactive Inc, Beijing, China) was used for immersive VR.	A double-blind, parallel-group, block-randomized, controlled trial	A total of 20 participants were blinded and randomized to VR group (n = 11) or e-learning group (n = 9).	● Ultrasound skills based on the OSAUS (Objective Structured Assessment on Ultrasound Skills).<br>● Hand-eye score based on Shapiro-Wilks Test questionnaire	● The VR group (n = 11) scored a significantly higher Objective Structured Assessment on Ultrasound Skills score (143 [95% confidence interval {CI}, 135 to 151]) compared with the e-learning group (n = 9; 126 [95% CI, 113 to 138]; mean difference, 17 points [95% CI, 4 to 30]; p < 0.01).<br>● The hand-eye score was higher in the VR group, although not at a significant level (mean difference, 3 points [95 % CI, -3 to 9]; p = 0.32).<br>● Ninety-one percent of the VR group wanted more VR learning.	2b
Andersen *et al.*, 2021 [63]	Denmark	to examine the impact of adding immersive VR (IVR) training to a course in ultrasound	A VR-ready computer (specifications: i7-6800K, 64GB RAM, GTX 1080	A double blinded, randomized controlled	Medical students were randomly assigned to either IVR (n = 10) or no	● Proportion of successful peripheral venous	● The proportion of successful peripheral venous cannulations was significantly higher in the IVR group (p ≤ .001). The proportions of successful cannulations were significantly	2b

Author (year)	Country	Purpose	Virtual simulator/ platform /system	Study design	Included population	Outcomes measurements	Main findings	Kirkpatrick level
		(US)-guided peripheral venous cannulation.	8GB) was used to run the IVR simulator (HTC Vive System, Taipei, Taiwan; VR USGIVA 1.0 VitaSim, Odense, Denmark).	study	further training (n = 9).	cannulations on a phantom.<br>● The proportion of surface punctures on the phantom and procedure time.	higher in the IVR group compared to the control group for the 1st and 2nd PVC ($p = .011$, $p = .023$), but not for the 3rd PVC ($p = .087$).<br>● Similar results were found for the proportion of surface punctures (1st: $P \leq .001$, 2nd: $P = .001$, and 3rd:$p = .114$).<br>● No significant differences in procedure times were found between the groups.<br>● 73% of all cannulations were successful in the intervention group, compared to 22% in the control group.	
Frendø *et al.*, 2021 [64]	Denmark	To explore learning curves in VR simulation-based training (SBT) of Cochlear Implant (CI) surgery and transfer of skills to a 3D-printed model.	The Visible Ear Simulator (VES), a free software for temporal bone VR simulation.<br>A Geomagic Touch haptic control device (3D Systems, Rock Hill, SC, USA) provides force feedback.	A prospective, single-arm trial	Twenty-four novice medical students	Pre- and post-training test, in which surgical performance measured by a seven-item modified Cochlear Implant Surgery Assessment Tool (CISAT) score.	● Learning curves were highly individual with primary performance improvement initially, and small but steady improvements throughout the 18 procedures.<br>● CI VR simulation performance improved 33% ($p < 0.001$).<br>● Insertion performance on a 3D-printed temporal bone improved 21% ($p < 0.001$), demonstrating skills transfer.	2b
Frendø *et al.*, 2021	Denmark	To develop and gather validity evidence for a novel Cochlear	Visible Ear Simulator (VES) version 3.0.<br>A Geomagic Touch	A prospective study	Fifteen CI-surgeons, nine trained novices and 11 medical	Performance rated by three blinded experts	● The CISAT significantly discriminated between the three groups ($p < 0.001$). The generalizability coefficient was 0.76 and most	2b

Author (year)	Country	Purpose	Virtual simulator/ platform /system	Study design	Included population	Outcomes measurements	Main findings	Kirkpatrick level
[65]		Implant Surgery Assessment Tool (CISAT) for assessment of cochlear implant (CI) surgery, including VR CI surgery training.	device (3D Systems, Rock Hill, SC, USA) delivers haptics for force-feedback.		students as true novice group	surgical	of the score variance (53.3%) was attributable to the participant and only 6.8% to the raters.<br>● When exploring a standard setting for CI surgery, the contrasting groups method suggested a pass/fail score of 36.0 points (out of 55).	
Petersen *et al*., 2021 [67]	Denmark	To investigate whether pretraining of basic skills in virtual vitreoretinal surgery affected the performance curve when proceeding to procedure-specific modules.	The EyeSi Surgical Simulator (software version 3.4, VRmagic GmbH, Manheim, Germany)	A prospective, randomized, controlled, two-centre study	A total of 68 medical students equally randomized into two groups. Group 1 performed pretraining of basic skills until they reached plateau on the performance curve.	● Accumulated time used to complete the procedure-specific modules.<br>● Starting scores and plateau levels of the procedure-specific modules.	● The participants in Group 1 used a median time of 88 minutes to reach plateau in the basic skills modules but did not differ from Group 2 in time to reach plateau on the procedure- specific modules (183 min versus 210 min, p = 0.40) or in the amplitude of plateau.<br>● Group 1 and 2 differed significantly in the starting score of ILM peeling level 3 (0 (0-0) versus 3.5 (0-75), p = 0.03).	2b
Bouaoud *et al*., 2021 [70]	France	To assess DIVA as an educational tool for craniofacial trauma for undergraduate medical students.	DIVA (Data Integration and Visualization in Augmented and Virtual Environments, Pasteur Institute, Paris), a software allowing the	A intervention Study	Fourth-year undergraduate medical students (n=50)	An 8-item questionnaire assessing satisfaction, potential benefit, ease of use and tolerance.	● High degrees of satisfaction (98%) without specific tolerance issues (86%) were reported.<br>● The potential benefit in a better understanding of craniofacial trauma using VR was reported by almost all students (98%).	1

**Author (year)**	**Country**	**Purpose**	**Virtual simulator/ platform /system**	**Study design**	**Included population**	**Outcomes measurements**	**Main findings**	**Kirkpatrick level**
			straightforward visualization of CT-scans in a 3D VR environment.					
Nakai *et al.*, 2021 [72]	Japan	To share experience with conducting anatomy lectures for medical student using a VR workspace.	A VR workspace (Spatial) using VR headset (Oculus Quest 2, Menlo Park, CA)	A pilot study	Thirty medical students	A post lecture survey to gain feedback from the students.	Many of our participants expressed that having access to their course material from anywhere and anytime via a virtual space, and being able to manipulate anatomical structures by moving and modifying them provided the student with a strong advantage.	1
Takata *et al.*, 2021 [75]	Japan	To evaluated operator 3D motion sickness while using a VR simulator and assessed whether it can be reduced by repeating the training.	The manipulation model (Intuitive Surgical) with the da Vinci robot.	A prospective observational study	Thirty medical students were randomized into the trained or non-trained group.Fifteen students were enrolled in the trained group.	● Performance time scoring the da Vinci robot's operating ability.<br>● Motion sickness determined using the Visual Analog Scale and Simulator Sickness Questionnaire (SSQ) before and after the training.	● Manipulation time significantly improved after training compared to before training (293.9 ± 72.4 versus 143.6 ± 18.4 s; $p < 0.001$).<br>● Although motion sickness worsened after each training session, it gradually improved with continuous practice with the VR simulator.<br>● SSQ subscores showed that the VR simulator induced nausea, disorientation, and oculomotor strain, and oculomotor strain was significantly improved with repeated training.	2b

Author (year)	Country	Purpose	Virtual simulator/ platform /system	Study design	Included population	Outcomes measurements	Main findings	Kirkpatrick level
Rothlind *et al.*, 2021 [77]	Sweden	To explore a learner perspective on the educational use of a virtual patient system designed to contribute to training in cultural competence in a primary care context.	BSA-sim (virtual patients with refugee backgrounds), an online virtual patient system.	A intervention Study	Twelve informants including eight medical students, three residents, and one specialist in family medicine.	Physicians and medical student s were invited to test the cases and participate in an interview about their experience.	● The analysis generated the theme virtual patients might help improve cultural competence in physicians and medical students by complementing knowledge gained through the informal curriculum.<br>● Informants at different educational levels found the educational use of a virtual patient system could be suitable as a tool for introducing the topic and for reflecting on one's own consultations. It could also compensate for the predominant informal manner of learning cultural competence, described by informants.	1
Arents *et al.*, 2021 [83]	The Nether-lands	To investigate whether adding a 360° VR video to the internship curriculum leads to an improvement of long-term recall of specific knowledge on a gentle Caesarean Sections (gCS) and on general obstetric knowledge.	A 360° VR video of a gentle caesarean section shown on a VR headset (Oculus Go).<br>.	A comparative Study	A total of 89 medical students (41 in the 360° VR video group and 48 in the conventional study group).	● An open questionnaire assessing knowledge on the gCS.<br>● A multiple-choice questionnaire assessing knowledge on general obstetrics.<br>● Post-survey.	● Watching the 360° VR video did not result in a difference in either specific or general knowledge retention between the intervention group and the conventional study group.<br>● 83.4% of the 360° VR video-group reported that more videos should be used in training to prepare for surgical procedures.<br>● In the 360° VR video-group, 56.7% reported side effects like nausea or dizziness.<br>● Students in the 360° VR video-group stated less often ($p = 0.04$) that they would have liked to attend more CSs in-person as compared to the conventional study group.	2b
Beulens *et al.*,	The Nether-	To understand the influence of proctored	The Robotix Mentor simulation system	A prospective	A total of 70 participants	● Pre- and post-intervent	● All the participants showed a significant improvement in their dexterity skills (the	2b

Author (year)	Country	Purpose	Virtual simulator/ platform /system	Study design	Included population	Outcomes measurements	Main findings	Kirkpatrick level
2021 [84]	lands	guidance versus simulator generated guidance (SGG) on the acquisition dexterity skills in novice surgeons learning RAS (robot assisted surgery).	designed by 3D systems.	non-blinded 3-arm randomised controlled trial	including 49 medical students, 13 PhD-students, and 5 surgical residents were assigned to three different interven- tion groups and received a different form of guidance:1) proctored guidance, 2) simulator generated guidance, 3) no guidance, during training on VR simulator.	ion vesicourethral anastomosis simulation exercise scores.<br>● Participant satisfaction questionnaire .	ability to perform RAS) after the training. There was no significant difference in the improvement of surgical skills between the three different intervention groups.<br>● The proctored guidance group reported a higher participant satisfaction compared to the simulator-generated guidance group, which could indicate a higher motivation to continue the training.<br>● Novice surgeons significantly increase their dexterity skills in RAS after a short time of practicing on simulator.	
García-Seoane *et al.*,2021 [87]	Spain	To describe the creation, administra-tion, and development of a computer-based case simulation Objective Structured Clinical Examination (CCS-OSCE) test.	A self-developed CCS-OSCE based on online educational platforms including Moodle, Sakai and Blackboard.	A intervention study	A total of 2829 medical students from 13 medical school in their sixth year of study	A satisfaction survey	Students evaluated as adequate or better the prior information received (70%), the test day organization (83%), the preparation received during their degree (67%), the prior knowledge they had acquired (88%), and the type of medical problems presented compared to the content seen during the degree (76%). Most considered the test to be a good learning experience (75%).	1

**Author (year)**	**Country**	**Purpose**	**Virtual simulator/ platform /system**	**Study design**	**Included population**	**Outcomes measurements**	**Main findings**	**Kirkpatrick level**
Gunn *et al.*, 2021 [88]	Australia	To report on the student experience of the integration of VR education for both medical imaging (MI) and radiation therapy (RT) students in learning computed tomography (CT) scanning.	The VR CT Sim software	A intervention Study	MI students (n = 28) and RT students (n = 38)	A post-survey evaluating students' perceived confidence in performing diagnostic and planning CT scans in the clinical environment.	68% of students agreed or strongly agreed to the addition of VR CT simulation helping in the learning of CT.	1
Han *et al.*, 2021 [93]	Korea	To develop a VR-based Neurological Examination Teaching Tool (VRNET) and evaluated its usefulness in in teaching neurological examinations for the medical students.	The self-developed VRNET, a VR program that performs neurological tests using Oculus Rift, a popular VR head set developed by Oculus VR (a division of Facebook) (Oculus VR, SF, USA).	A prospective, randomized, single-blind study	A standardized patient (SP) group comprised 39 senior (fourth year) medical students and the SP with VRNET group had 56 students.	● Neurologic Physical Exam (NPE) score.<br>● A five-point scale indicating realness and satisfaction with care experiences with SPs.	● There were no statistical differences in VRNET's realness and student satisfaction between the SP and SP with VRNET groups.<br>● A statistically significant difference was found in the NPE score ($p = 0.043$); the SP with VRNET group had higher NPE scores (3.81 ± 0.92) than the SP group (3.40 ± 1.01).	2b

**Author (year)**	**Country**	**Purpose**	**Virtual simulator/ platform /system**	**Study design**	**Included population**	**Outcomes measurements**	**Main findings**	**Kirkpatrick level**
O'Connor *et al*., 2021 [96]	Ireland	To pilot an immersive three-dimensional (3D) virtual radiography simulation tool in an undergraduate Radiography curriculum and to retrieve user feedback.	3D VR software (Virtual Medical Coaching Ltd) and hardware, which consisted of HTC Vive Pro™ headsets and hand controllers.	A intervention study	First year radiography students (n = 105)	An online survey was used to evaluate students' perceptions of the VR radiography simulation software as an educational tool.	● Most respondents (58%) reported enjoying VR simulation, whilst some felt indifferent towards it (27%).<br>● Ninety-four percent would recommend this tool to other students.<br>● The mean length of time it took for students to feel comfortable using the technology was 60 min (10–240 min).<br>● Most respondents (58%) desired more VR access.<br>● Students attributed enhanced confidence in the areas of beam collimation (75%), anatomical marker placement (63%), centring of the X-ray tube (64%) and exposure parameter selection (56%) to their VR practice.<br>● Many students (55%) advocated the use of VR in formative or low stakes assessments.	1
Sattar *et al*., 2021 [98]	Pakistan	To investigate the effects of design parameters on the user experience of VR medical training.	The VR application app (Medical Realities) available on both Android and iOS operating systems.	A quantitative study	A total of 87 final year students, each of who was given to experience laparoscopy operation in text, video and virtual reality-based learning methodologies.	User experience and usefulness was assessed against a pre-validated scale and compared with the three learning	User experience and usefulness of VR was better than others ($p<0.05$).	2a

Author (year)	Country	Purpose	Virtual simulator/ platform /system	Study design	Included population	Outcomes measurements	Main findings	Kirkpatrick level
						methodologies.		
Khundam *et al.*, 2021 [99]	Thailand	To investigate the differences in usability between using hand tracking and controllers during the VR intervention for intubation training for medical students	The case study of VR applications was self-developed using OVRInput and OVRHand Class Reference on Unity3D.	A cross-sectional study	Forty-five medical undergraduates were divided into 3 groups: video only, video with VR controller training, and video with VR hand tracking training, each of which contained 15 students.	● Pre-test, post-test, and practice scores.<br>● The System Usability Scale (SUS) and User Satisfaction Evaluation Questionnaire (USEQ) .	● The overall learning outcomes of both VR groups were better than those of the video group.<br>● The post-test scores (p=0.581) and practice scores (p=0.168) of both VR groups were not significantly different.<br>● No significant between-group differences were found in the SUS scores (p=0.588) or in any aspects of the USEQ scores.	2b
Mansoory *et al.*, 2021 [100]	Iran	To compare the effectiveness of lecture method and virtual reality-based serious gaming (VRBSG) method on students learning outcomes about the test approach to coma.	The game stages were designed by Unity game engine, ZBrush, Blender, V-Ray and 3ds Max software.	A randomized trial pretest-posttest design	Medical students were divided into VRBSG and lecture groups (each group consisting of 25 students)	● Students' learning outcome was measured with a 10-item test.<br>● Serious game usability scale was used to evaluate the usability of the serious game.	● Students' familiarity with e-learning and VRBSG was low. The mean usability of a VRBSG was 126.78 ± 10.34 out of 150.<br>● The majority of students were eager to be instructed through VRBSG.<br>● The mean score of learning outcomes in the VRBSG group was significantly higher than the lecture group (t = - 2.457, p = 0.019).	2b

Author (year)	Country	Purpose	Virtual simulator/ platform /system	Study design	Included population	Outcomes measurements	Main findings	Kirkpatrick level
Berg & Steinsbekk, 2021 [103]	Norway	To investigate whether group self-practice of systematic clinical observation using the airway, breathing, circulation, disability and exposure (ABCDE) approach in a multiplayer, immersive, interactive VR application provided a non-inferior learning outcome compared to practicing with physical equipment.	The self-developed VirSam ABCDE application with hired help for programming in Unity.VR equipment consisting of an head mounted display and hand controllers (Oculus Rift S or Oculus Quest; https://www.oculus.com/).	A non-inferior parallel-group randomized controlled trial	All 289 first-year medical and nursing students (70 medical 219 nursing) were randomly allocated to practice ABCDE in a fully immersive, interactive, multiplayer VR application (the VR group, n=146) or with physical equipment (the TP group, n=143).	● A practical test.<br>● Participants' knowledge and performance of the ABCDE approach and their experience with the teaching session.	● Group self-practice of the ABCDE approach in multiplayer, immersive, interactive VR application was non-inferior to practice with physical equipment.<br>● For other outcomes, the results were mostly similar between the groups.	2b
Birrenbach *et al.*, 2021 [105]	Switzerland	To explore the short- and long-term effectiveness of a fully immersive VR simulation versus a traditional learning method regarding a COVID-19-related skill set and media-specific variables influencing training outcomes.	VR simulation (CVRSB (Covid19 – VR strikes back) module, version 1.1.6, software platform, developed by ORamaVR (Heraklion, Crete, Greece) and the Oculus Rift S head mounted device and hand controllers (Facebook Inc., Menlo Park, California, USA).	A prospective, randomized controlled pilot study	Medical students n=29 (intervention VR training, n=15, vs control video-based instruction, n=14)	● Performance of taking a nasopharyngeal swab sample assessed using a 17-item checklist.<br>● User Satisfaction Evaluation Questionnaire (USEQ).	● After training, the VR group performed significantly better in taking a nasopharyngeal swab, scoring a median of 14 out of 17 points (IQR 13-15) versus 12 out of 17 points (IQR 11-14) in the control group,p=.03.<br>● With good immersion and tolerability of the VR simulation, satisfaction was significantly higher in the VR group compared to the control group (median score of USEQ 27/30, IQR 23-28, vs 22/30, IQR 20-24, in the control group; p=.01).	2b
